# Individualized microbiotas dictate the impact of dietary fiber on colitis sensitivity

**DOI:** 10.1186/s40168-023-01724-6

**Published:** 2024-01-05

**Authors:** Erica Bonazzi, Alexis Bretin, Lucile Vigué, Fuhua Hao, Andrew D. Patterson, Andrew T. Gewirtz, Benoit Chassaing

**Affiliations:** 1 INSERM U1016, Team “Mucosal Microbiota in Chronic Inflammatory Diseases”, CNRS UMR10 8104, Université Paris Cité, Paris, France; 2 Institute for Biomedical Sciences, Centre for Inflammation, Immunity and Infection, Digestive Disease Research Group, Georgia State University, Atlanta, GA USA; 3 INSERM U1016, Team “Robustness and Evolvability of Life”, CNRS UMR10 8104, Université Paris Cité, Paris, France; 4 Department of Veterinary and Biomedical Sciences, Center for Molecular Toxicology and Carcinogenesis, Pennsylvania State University, University Park, PA USA

**Keywords:** Microbiota, Dietary fiber, Inflammation, Personalized response

## Abstract

**Background:**

The observation that the intestinal microbiota is  central in the development of IBD suggests that dietary fiber, the microbiota’s primary source of nourishment, could play a central role in these diseases. Accordingly, enriching diets with specific soluble fibers remodels microbiota and modulates colitis sensitivity. In humans, a recent study suggests that the microbiota of select IBD patients might influence the impacts they would experience upon fiber exposure. We sought here to define the extent to which individual microbiotas varied in their responsiveness to purified soluble fiber inulin and psyllium. Moreover, the extent to which such variance might impact proneness to colitis.

**Results:**

We observed a high level of inter-individual variation in microbiota responsiveness to fiber inulin and psyllium: while microbiotas from select donors exhibited stark fiber-induced modulation in composition, pro-inflammatory potential, and metabolomic profile, others were only minimally impacted. Mice transplanted with fiber-sensitive microbiomes exhibited colitis highly modulated by soluble fiber consumption, while mice receiving fiber-resistant microbiotas displayed colitis severity irrespective of fiber exposure.

**Conclusion:**

The extent to which select soluble fibers alter proneness to colitis is highly influenced by an individual's microbiota composition and further investigation of individual microbiota responsiveness toward specific dietary fiber could pave the way to personalized fiber-based intervention, both in IBD patients and healthy individuals.

Video Abstract

**Supplementary Information:**

The online version contains supplementary material available at 10.1186/s40168-023-01724-6.

## Introduction

Dietary fibers are increasingly appreciated to be a central component of an optimal human diet, conferring beneficial impacts on intestinal and metabolic health [1]. Being largely comprised of plant cell walls, dietary fibers harbor various molecular structures that impact their physicochemical properties, including viscosity and solubility [2]. Fibers with high degrees of solubility are readily fermented by intestinal microbes, providing short-chain fatty acids (SCFA) [2], and contributing to the ability of soluble fibers to protect against metabolic syndrome in both animal models and human clinical studies [3]. In contrast, the impacts of fibers on inflammatory bowel disease (IBD) are less clear. Epidemiological studies have suggested that consumption of fiber-rich diets is associated with a reduced incidence of IBD, albeit numerous confounding factors can be at play [4]. Yet there are reports that once the disease is established, IBD patients experience intolerance to fermentable fiber-rich foods while some IBD patients suspect it to trigger disease flares [5, 6]. Parallel complexity can be found in animal studies. For example, feeding mice a low-fiber diet rather than a diet naturally rich in fibers predisposes them to severe DSS-induced colitis yet enriching such low-fiber diets with the highly soluble fiber inulin further exacerbates such colitis. In contrast, enriching such diets with the medium soluble fiber psyllium provides strong protection in the DSS-induced and T cell transfer colitis models [7, 8]. Thus, the impacts of fibers are likely fiber- and context-specific.

A recent clinical study by Armstrong and colleagues suggests that the impacts of fibers may be greatly influenced by one’s individual microbiota composition. Specifically, they found that dysregulated fermentation of inulin could trigger pro-inflammatory response via activation of NLRP3 and TLR2 pathways in a subset of IBD patients lacking specific fermentative microbe activity [6]. Hence, we hypothesized that the extent to which purified soluble fiber inulin and psyllium impact colitis susceptibility, either detrimentally or beneficially, is highly variable between individuals as a result of microbiota heterogeneity. We tested this hypothesis via an in vitro microbiota modeling system that enabled the assessment of microbiota composition and functional readouts. We observed a high level of inter-individual variance in the extent to which microbiotas were altered by exposure to inulin or psyllium. Such responses predicted the extent to which the severity of colitis was altered by select fiber consumption in mice transplanted with these microbiotas. These results support the emerging hypothesis that the extent to which soluble fibers are beneficial/detrimental is highly microbiota-dependent and individual-specific.

## Results

### Impacts of fibers on the composition of human intestinal microbiotas ex vivo are highly donor-specific

We hypothesize here that the extent to which purified soluble fibers inulin and psyllium impact colitis susceptibility, either detrimentally or beneficially, is highly variable between individuals as a result of microbiota heterogeneity. We investigated this hypothesis via the in vitro microbiota modeling system MiniBioReactor Array (MBRA), a dynamic system allowing parallel anaerobic human microbiota cultures (Figure S1A). MBRA chambers were filled with BRM medium (Table S2) before being inoculated with fecal microbiota from six healthy donors and allowed to stabilize for three days, as previously reported [9] (Figure S1B). Equilibrated MBRA microbiota were collected and compositionally analyzed by 16S rRNA gene sequencing, which showed that this system captures individualized microbiota in a highly reproducible manner (Figure S2A). Taxonomical analysis further confirmed this notion, with differences being observed at the class level such that differences between chambers inoculated from the same donor were far smaller than those derived from other subjects/donors (Figure S2B and S2C).

**Fig. 1:**
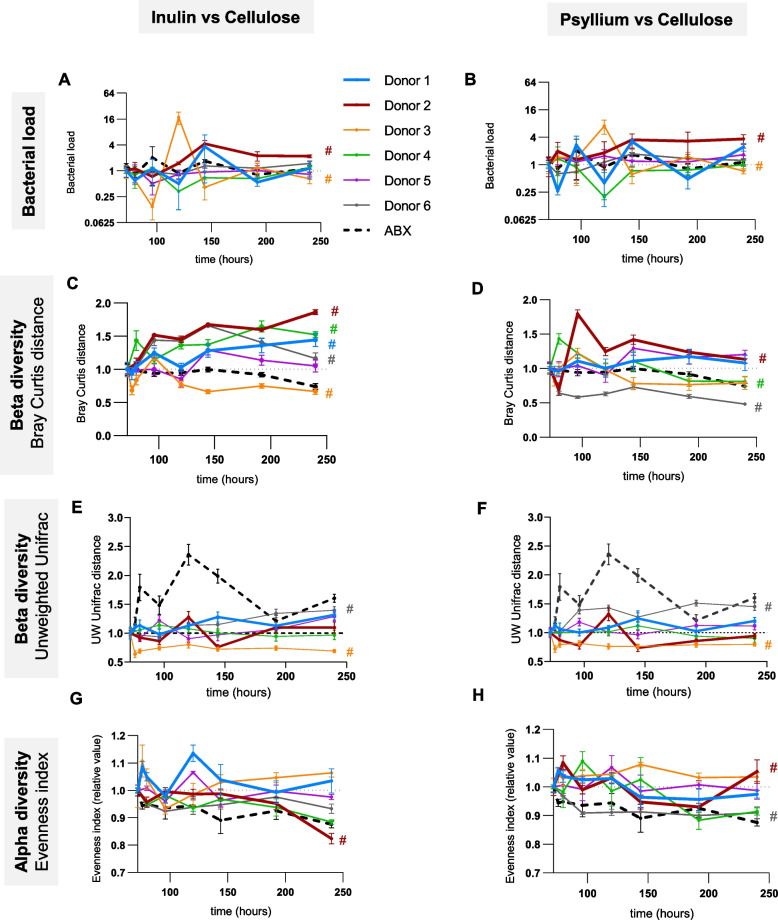
Inter-individual variations in fibers-induced microbiota composition alterations. The in vitro microbiota MBRA system was inoculated with fecal slurry from 6 healthy donors and stabilized for 72 h, at which point fiber treatment was applied using cellulose, inulin, or psyllium. **A**, **B** Bacterial DNA was extracted and 16S rRNA qPCR-based bacterial density quantification. For each donor, the bacterial load is expressed as a relative value for inulin-treated (**A**) or psyllium-treated (**B**) chambers compared to cellulose-treated chambers. Extracted DNA was subjected to Illumina-based 16S rRNA gene sequencing and **C**–**F** Beta diversity evolution was computed through the QIIME2 pipeline using the Bray–Curtis (**C**, **D**) or the Unweighted Unifrac (**E**, **F**) distance matrix. For each donor, the evolution of microbiota composition is represented using distances expressed as relative values for inulin-treated (**C**, **E**) or psyllium-treated (**D**, **F**) chambers compared to cellulose-treated chambers. **G**, **H** Alpha diversity evolution computed through the QIIME2 pipeline using the Evenness index. For each donor, the evolution of microbiota richness is represented using the Evenness index expressed as a relative value for inulin-treated (**G**) or psyllium-treated (**H**) chambers compared to cellulose-treated chambers. Donor 1 and donor 2 are represented in bolded in all the data related to the use of the MBRA system, since they were subsequently used to perform fecal microbial transplantation. Data are the means ± S.E.M (*N* = 3). Significance was determined using 2-way group ANOVA corrected for multiple comparisons with the Bonferroni test (# indicates *p* < 0.05) compared to the control group (cellulose-treated chambers). Color of the # sign corresponds to the donor for which statistical significance is reached

After the stabilization phase, MBRA microbiota were subsequently administered soluble fiber inulin or psyllium (0.02% w/v), or the insoluble fiber cellulose, which was used as a control (0.02% w/v) (Figure S1B). We initially measured the impact of soluble fiber exposure on microbiota density, (i.e., total bacterial load since volume was constant) using qPCR with universal 16S primers. The obtained data were displayed as the impact of either inulin (Fig. 1A) or psyllium (Fig. 1B), with cellulose-treated microbiota being used as a reference throughout the treatment phase. We found that microbiota density was only modestly impacted by soluble fibers in select donors (#2, *P* value 0.005; #3, *P* value 0.027) (Fig. 1A, B). In contrast, assessing microbiota composition via 16S rRNA gene sequencing revealed differences between donors in response to both inulin and psyllium (Fig. 1C–F). Specifically, using the weighted Bray–Curtis distance metric, we observed microbiotas from donors 2, 4, and 6 displayed profound (#2, *P* value < 0.0001; #4, *P* value < 0.0001, #6, *P* value < 0.0001) changes in composition upon exposure to inulin or psyllium, relative to cellulose-treated control microbiota (Fig. 1C, D). Unweighted UniFrac analysis revealed inulin- and psyllium-induced compositional changes only for donors 3 and 6 (#3 and #6, *P* value < 0.0001), suggesting that inulin and psyllium impact on microbiota composition of the other donors occur through modulation of relatively abundant members (Fig. 1E, F). Analysis of microbiota alpha diversity revealed that inulin and psyllium had modestly impacted microbiota richness and evenness, and did so only in select donors (#2, *P* value < 0.0001; #6, *P* value < 0.0001 for psyllium). Antibiotic-treated microbiota, used as a positive control of microbiota disturbance, further highlights that at the concentration used, inulin and psyllium only modestly impacted microbiota composition (Fig. 1G, H and data not shown).

### Dietary fibers-induced modulation of the intestinal microbiota pro-inflammatory potential is highly donor-specific

**Fig. 2:**
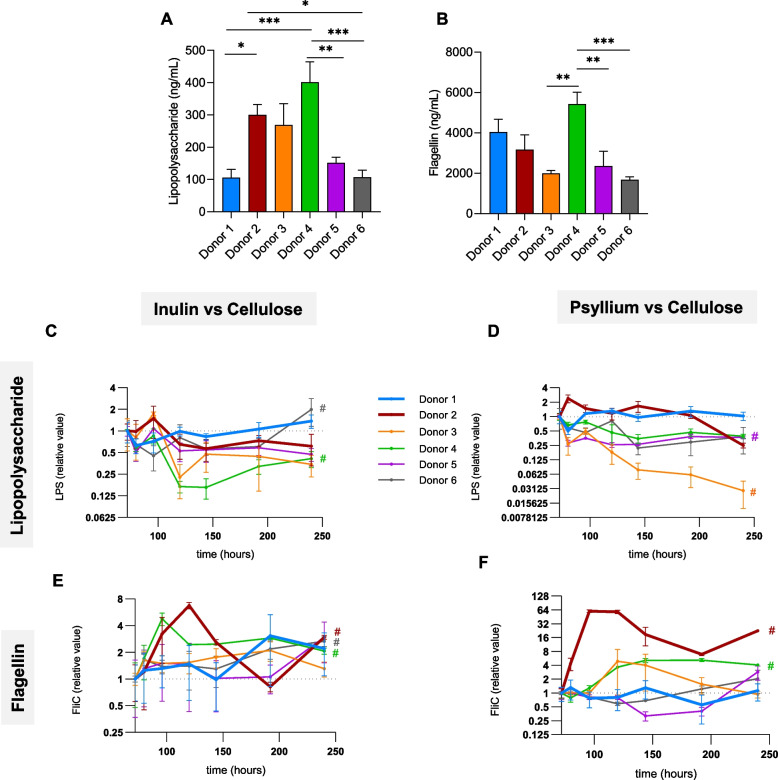
Inter-individual variations in fibers-induced modulation of microbiota pro-inflammatory potential. The in vitro microbiota MBRA system was inoculated with fecal slurry from 6 healthy donors and stabilized for 72 h, at which point fiber treatment was applied using cellulose, inulin, or psyllium. Microbiota-derived expression of pro-inflammatory molecules lipopolysaccharide (**A**, **C**, and **D**) and flagellin (**B**, **E**, and **F**) were quantified using HEK reporter cells expressing TLR4 or TLR5, respectively. **A**, **B** Microbiota-derived bioactive lipopolysaccharide (**A**) and flagellin (**B**) levels at the end of the stabilization period (72 h timepoint). **C**–**F** For each donor, the evolution of microbiota-derived bioactive lipopolysaccharide (**C**, **D**) and flagellin (**E**, **F**) levels are expressed as relative value for inulin-treated (**C**, **E**) or psyllium-treated (**D**, **F**) chambers compared to cellulose-treated chambers. Donor 1 and donor 2 are represented in bolded in all the data related to the use of MBRA system, since they were subsequently used to perform fecal microbial transplantation. Data are the means ± S.E.M (*N* = 3). In **A**, **B**, significance was determined using one-way ANOVA followed by a Tukey’s multiple comparison test, and significant differences were presented as follows: *p < 0.05, **p < 0.01, and ***p < 0.001. In **C**–**F**, significance was determined using 2-way group ANOVA corrected for multiple comparisons with the Bonferroni test (# indicates *p* < 0.05) compared to the control group (Cellulose-treated chambers). Color of the # corresponds to the donor for which statistical significance is reached

We next investigated the impact of soluble fibers on functional aspects of the MBRA microbiotas. Specifically, we assessed the impacts of purified inulin and psyllium on microbiota pro-inflammatory potential through the use of TLR4 and TLR5 reporter cells thus allowing quantification of bioactive levels of lipopolysaccharide (LPS) and flagellin, respectively. These microbiota-derived MAMPs (microbe-associated molecular patterns) appear to be the main microbiota-derived sources of innate immunity activation [10], and their levels were previously reported to correlate with the ability of a given microbiota to activate innate immune signaling that perpetuates chronic intestinal inflammation [11–13]. We observed that post-stabilization, MBRA levels of LPS and flagellin differed significantly between individual donors, supporting the suitability of the MBRA system to capture functional inter-individual differences (Fig. 2A, B). More importantly, the extent to which MBRA microbiota expression of LPS and flagellin was modulated by fiber exposure was highly individualized (Fig. 2C–F). More specifically, microbiota from donors 2 and 4 harbored bioactive levels of lipopolysaccharide and flagellin that were highly modulated by soluble fiber inulin and/or psyllium (#2, *P* value < 0.0001 for flagellin when exposed to both inulin and psyllium compared to cellulose; #4, *P* value: 0.012 for LPS when exposed to inulin and *P* value < 0.0001 for flagellin when exposed both to inulin and psyllium), while microbiota from donors 1 and 5 appear to have stable and fibre-independent MAMPs levels (Fig. 2C–F). Microbiota from donor 6 also harbored inulin-mediated modulation of LPS and flagellin loads (#6, *P* value 0.032 for LPS and *P* value 0.0028 for flagellin). Another functional readout, namely metabolomic analysis, found that microbiota from some donors produce various metabolites whose concentrations are modulated by soluble fiber, while microbiota from other donors were not impacted in their overall metabolite production between experimental groups (Figure S3 and S4). In particular, PCoA plots from donors 2, 3, 4, and 6 revealed modest differences in metabolite production following inulin or psyllium exposure, with *P* values of *P* = 0.030, *P* = 0.006, *P* = 0.029, *P* = 0.010, respectively. On the other hand, microbiota from donors 1 and 5 did not show a significant impact of inulin or psyllium exposure on their metabolite production (Figure S3). Hence, these results collectively indicate that microbiotas from distinct individuals have individualized sensitivity toward inulin and psyllium exposure.

### Multi-variables-based identification of fiber-sensitive and fiber-resistant microbiota

**Fig. 3:**
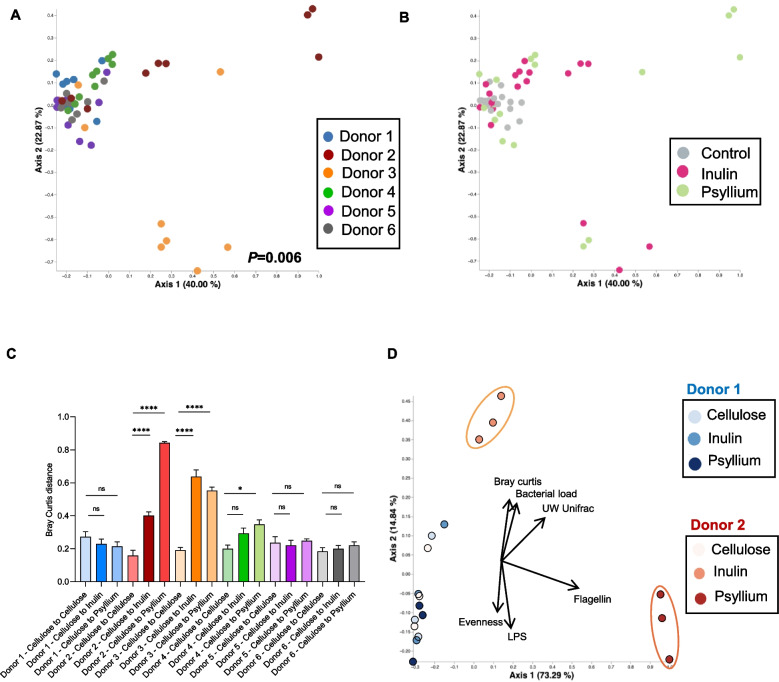
Identification of resistant and sensitive microbiota toward fiber exposure. Principal coordinates analysis (PCoA) of the Bray–Curtis distance computed on the following MBRA-based parameters determined 48 h after the initiation of fibers treatment are presented: bacterial load, beta diversity (Bray–Curtis and unweighted Unifrac distances), alpha diversity (Evennenss distance), lipopolysaccharide and flagellin bioactive levels. In **A**, **B**, all donors are included, and dots are coloured by donor (**A**) or by treatment (**B**). **C** Histogram representing the cellulose-cellulose, cellulose-inulin, and cellulose-psyllium Bray–Curtis distance for the various donors included. **D** Principal coordinates analysis (PCoA) of the Bray–Curtis distance computed on the above-listed parameters, with only donors 1 and 2 included. Vectors for the included variables are represented. Data are the means ± S.E.M. Significance was determined using one-way ANOVA followed by a Tukey’s multiple comparison test and significant differences were presented as follows: *p < 0.05, ****p < 0.0001

We next sought to develop an analytical approach that would take into account both sequencing and functional readouts to produce an output that reflected the overall extent to which an individual’s microbiota was altered ex vivo by dietary fiber. We performed principal coordinate analyses using Bray–Curtis distances, which were computed from a combination of compositional (beta diversity, alpha diversity) and functional (bacterial load, lipopolysaccharide, and flagellin bioactive levels) parameters. This approach confirmed that, overall, the extent of responsiveness to soluble fiber inulin and psyllium was highly individualized. More specifically, donors 1, 5, and 6 did not exhibit any treatment-based clustering, further indicating the minimal impact of inulin and psyllium exposure on these microbiotas, as assessed by the combination of species composition and bioactive levels of LPS and flagellin, a proxi of microbiota pro-inflammatory potential (Fig. 3A, B). This notion was confirmed by plotting the obtained Bray–Curtis distance, with the observation of similar distances, for these donors, between cellulose-cellulose, cellulose-inulin and cellulose-psyllium samples (Fig. 3C). In stark contrast, microbiota from donors 2 and 3 harbored a strong treatment-based clustering, with inulin and psyllium treatment driving specific clustering apart from cellulose-treated control chambers (Fig. 3A, B). Such plotting of the Bray–Curtis distances confirmed this observation, with the distance separating cellulose-treated and inulin- or psyllium-treated samples being significantly increased compared to the distance separating samples within the cellulose-treated group (Fig. 3C). PCoA plot of donors 1 and 2 samples confirmed a strong treatment-based clustering for donor 2, while donor 1 appeared fully resistant to soluble-fiber-induced disturbances (Fig. 3D). In alignment with data presented in Figs. 1 and 2, the vectorial analysis revealed that inulin effects on donor 2 were mostly related to modulation of bacterial load and composition, while psyllium effects on this donor were mostly related to modulation of microbiota pro-inflammatory potential, especially flagellin expression (Fig. 3D).

###  Comparison of fiber-sensitive vs. fiber-resistant microbiotas

Next, we examined the extent to which any features in baseline microbiota might predict their responsiveness to fiber. We first performed a metagenomic analysis and compared the composition of the three resistant donors to the composition of the two sensitive donors. We observed three differences that remained significant following correction for multiple comparisons, including *Faecalibacterium Prausnitzii*, associated with protection against IBD [14], which was only present in fiber-resistant microbiotas (Figure S5A). Subjecting metagenomic data to CAZymes (Carbohydrate-Active enZymes) comparison highlighted various differences, including increased abundance in many CAZymes in sensitive microbiota compared to resistant microbiota (Figure S5B), suggesting that sensitivity status might be linked to the ability of a given microbiota to ferment dietary fibers or mucin-derived glycans. Moreover, metagenomic-based analysis interestingly revealed that well-known fiber-fermenting microbes were present in both fiber-sensitive and fiber-resistance donors (Figure S5C), thus suggesting that basal difference in fiber-fermenting microbiota members is likely not the main actor driving sensitivity toward soluble fibers.

Metatranscriptomic analysis on MBRA samples collected during the treatment phase revealed a strong donor clustering (Figure S5D), suggesting the suitability of the MBRA system to keep interindividual variations in microbiota gene expression. Such metatranscriptomic analysis nonetheless revealed various pathways that are significantly impacted by inulin and/or psyllium in a given resistant donor (Donor 1) compared to a given sensitive donor (Donor 2) (Figure S5E). Hence, while discerning whether any of these differences truly relate to a microbiota’s degree of fiber sensitivity will require further studies, we next sought to investigate the in vivo relevance of the MRBA-based assessments of inulin- and psyllium-sensitivity using donors 1 and 2 as fiber-resistant and fiber-sensitive donors, respectively.

### Transplant of fiber-sensitive, but not fiber-resistant microbiota, to germfree mice results in fiber-mediated modulation of colitis severity

Germ-free C57BL/6 mice were next administered fecal transplants from donor 1 (fiber-resistant) or donor 2 (fiber-sensitive) (Figure S6A). Following a 1-week microbiota stabilization period, recipient mice were administered compositionally-defined diets containing either cellulose, inulin, or psyllium as a fiber source. Nineteen days later, mice were subjected to a one-week course of DSS exposure, thus enabling the investigation of both donor-dependent and fiber-dependent modulation of intestinal inflammation susceptibility (Figure S6B). Fecal samples collected throughout the above-described in vivo experimentation were used to evaluate the longitudinal impact of fiber exposure on microbiota load, composition, and pro-inflammatory potential, assessed through quantification of bioactive levels of fecal LPS and flagellin. While no major effects were observed on fecal bacterial density throughout the experimentation (Figure S7A–D), soluble fiber consumption strongly impacted microbiota composition, as revealed by Bray–Curtis distance analysis (Figure S8A–D). This approach indeed revealed that upon consuming an inulin- or psyllium-enriched diet, mice colonized with either donor 1 or donor 2 microbiota experienced strong alteration in their microbiota composition (Figure S8A–D). Moreover, we observed that microbiota composition behaved very similarly in mice colonized with either donor 1 or donor 2 microbiota, suggesting that ex vivo fiber responsiveness did not associate with impacts in this beta-diversity parameter. Yet, fiber impacts on alpha diversity were indeed donor-dependent in that we observed an increase of microbiota evenness in mice colonized with donor 1 (fiber-resistant) and treated with either type of fiber (Figure S8E–H), while mice colonized with donor 2 (fiber-sensitive) microbiota harbored a significant reduction in such parameter during the pre-DSS phase (Figure S8E–H). In light of these results, we next performed microbiota taxonomical analysis on day 19, prior to DSS exposure (Figure S9A, B). Such an analysis importantly revealed an increased relative abundance of *Bifidobacteriaceae* in inulin-fed mice transplanted with donor-resistant microbiota (9.11% ± 1.77% in inulin-fed mice versus 0.69% ± 0.29% in cellulose-fed mice, Figure S9A). Bacteria belonging to this family are known for their ability to ferment inulin with the downstream production of SCFA [15], and they were almost absent in mice transplanted with donor-sensitive microbiota (1.02% ± 0.15%, Figure S9B), suggesting that this SCFA producer can be involved in mediating fiber resistance. Finally, microbiota pro-inflammatory potential assessment showed that both lipopolysaccharide and flagellin bioactive levels were modulated by soluble fiber consumption, as expected [16], but without a clear donor effect (Figure S10), contrary to above described MBRA-based observations. Hence, this importantly suggests that the MBRA system appears more suitable to depict microbiota pro-inflammatory potential modulation following soluble fiber exposure. This observation is likely related to the fact that such a system assesses direct interaction between microbiota and fiber, without any host-mediated effect, unleashing microbiota’s responsiveness to a given dietary factor.

**Fig. 4:**
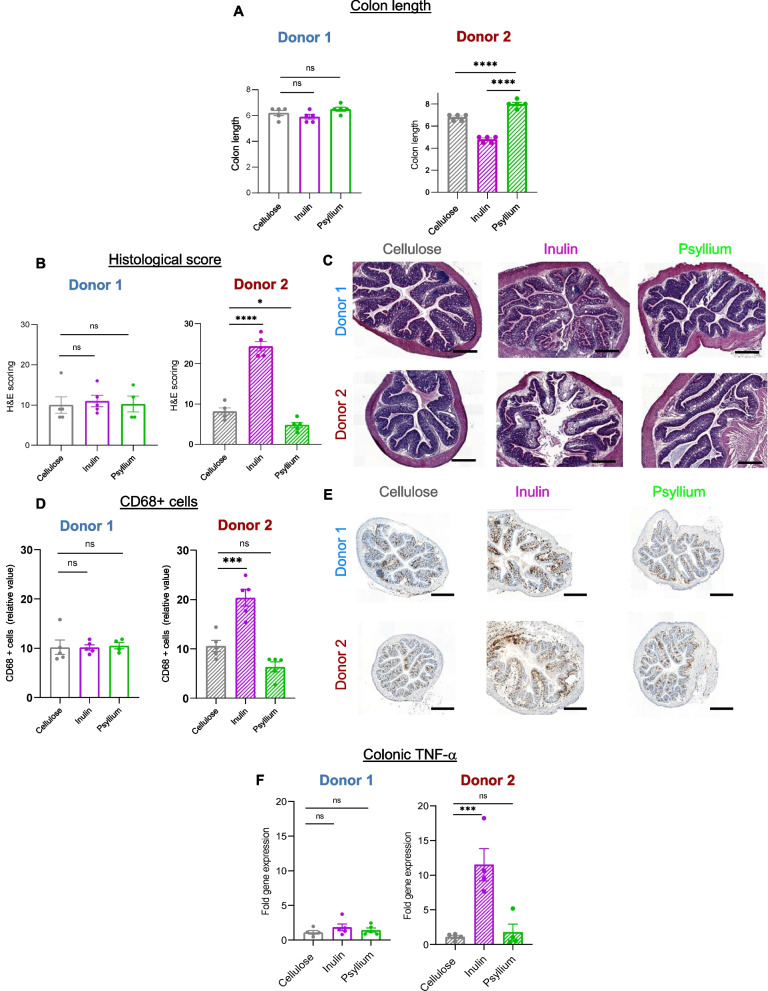
Fecal microbial transplantation reproduces fiber sensitivity status and drives individualized colitis susceptibility. **A**–**F** Upon arrival, germ-free WT mice undergo fecal microbial transplantation with fecal suspension from donor 1 (*fibers-resistant*) or donor 2 (*fibers-sensitive*) (*N* = 15 mice per donor). After 7 days of microbiota stabilization, mice were subsequently divided into three experimental groups and exposed to either cellulose- (grey), inulin- (purple), or psyllium- (green) supplemented diets for 25 days (*N* = 5 mice per experimental group). On day 19 and for 6 days, Dextran sulfate sodium was added to the drinking water (2.5% w/v) to induce intestinal inflammation. **A** Following euthanasia, colon lengths were measured. **B**, **C** Colonic sections were subjected to hematoxylin and eosin staining, and histological scoring of inflammation was performed (**B**). Representative images are presented in **C**. Bar = 100 μm. **D**, **E** Colonic sections were stained for CD68 monocyte marker, and 15 crypts were randomly selected per animal in order to determine the number of CD68 + cells per crypt (**D**). Representative images are presented in **E**. Bar = 400 μm. **F** Colonic mRNAs were extracted, and q-RT-PCR was used to evaluate the TNF-α pro-inflammatory cytokine expression level reported. Data are presented as relative values compared to the cellulose-treated group, defined as 1. Data are the means ± S.E.M, with individual data points being represented (*N* = 3). Significance was determined using a one-way ANOVA followed by Tukey’s multiple comparison test and significant differences were recorded as follows: *p < 0.05, ***p < 0.001, and ****p < 0.0001

We next investigated phenotypical consequences for the recipient mice colonized with either resistant or sensitive microbiota. Examination of body weight as a general indicator of health did not reveal differences induced by either the microbiota donor source or the fiber type being consumed during a relatively short period of time (Figure S11). However, the extent to which inulin and psyllium altered the severity of DSS colitis was highly donor-dependent and aligned with assessments of individual fiber sensitivity determined in the MBRA. Indeed, assessment of colitis severity by well-established indicators, namely colon length and histopathological scoring (Fig. 4A–C), both indicated that mice colonized with microbiota from donor 1 (fiber-resistant) exhibited similar severity of colitis irrespective of which fiber they consumed. In stark contrast, these readouts indicated that mice colonized with donor 2 microbiota (fiber-sensitive) exhibited protection against colitis when fed psyllium and exacerbated colitis when fed inulin, thus mimicking the impacts of these fibers on colitis in conventionally colonized mice [17]. CD68 + cell staining also revealed a fiber-dependant modulation of colitis in donor 2-colonized mice, with a detrimental impact of inulin and protection conferred by psyllium consumption, while donor 1-colonized mice developed colitis that was not modulated by soluble fiber inulin or psyllium consumption (Fig. 4D, E). Lastly, quantification of a central marker of chronic intestinal inflammation in both human and mice models, namely TNF-α cytokine, revealed a more than tenfold increase in mice colonized with a fiber-sensitive donor (donor 2) receiving an inulin-enriched diet, while mice colonized with fiber-resistant donor (donor 1) lacked such inulin-mediated effect (Fig. 4F). Altogether, these observations importantly revealed that microbiota identified as being fiber-sensitive, through our MBRA screening pipeline, was sufficient to drive inulin- and psyllium-modulated colitis, while microbiota identified as being fiber-resistant was not driving any fiber-modulated colitis.

## Discussion

While consumption of dietary fiber is widely associated with health-promoting effects, underly mechanisms of action remain unclear. Increasing evidence suggests that the intestinal microbiota could be a central actor in driving dietary fiber beneficial effects [16, 18]. Mainly categorized as soluble and insoluble fiber, depending on their structure and biochemical properties, the impact of soluble fiber is indeed thought to be mediated by intestinal microbes through the production of beneficial metabolites, such as short-chain fatty acids (SCFA), while the impact of insoluble fiber is mostly related to their ability to provide bulk and to modulate intestinal transit [2]. While consumption of soluble fiber, such as inulin and psyllium, appears associated with health in large epidemiological studies [19–21], mice studies interestingly revealed that inulin supplementation holds the potential to exacerbate colitis while psyllium supplementation provides strong protection in both DSS and T-cell transfer colitis models [17]. Hence, the impact of fiber on intestinal health is likely compound- and context-specific, perfectly aligning with the complex observation that while epidemiological studies suggest that consumption of fiber-rich diets correlates with a reduced incidence of IBD [4], numerous clinical studies suggest that, once the disease is established, some IBD patients experience intolerance to fermentable fiber-rich foods with disease flares [5, 6]. For example, Armstrong and colleagues recently reported that dietary β-fructan can induce pro-inflammatory cytokinic response in a subset of IBD patients who appears to lack select fermentative microbe activities [6].

Our study presented herein aimed to better understand the highly heterogeneous impacts of soluble dietary fiber inulin and psyllium on individual intestinal microbiotas from healthy subjects. For this purpose, we used a high-throughput in vitro human microbiota modeling system (MBRA), revealing that only select microbiota was impacted by soluble fiber inulin and psyllium supplementation, while other microbiota were fully resistant to fiber-mediated modulation. Moreover, fiber-sensitive microbiota was observed to be sufficient to drive soluble fiber-mediated modulation of intestinal inflammation when transplanted into germ-free mice, while mice colonized with fiber-resistant microbiota displayed colitis severity irrespective of dietary fiber content. More specifically, in mice transplanted with fiber-sensitive microbiota, DSS-induced colitis was exacerbated by inulin supplementation, while protection against intestinal inflammation was observed in these recipient mice treated with purified psyllium.

In depth characterization of donors’ microbiota in search for taxonomical or functional features associated with fiber responsiveness was performed through metagenomic analysis. Such an approach revealed various differences, including the presence of *Faecalibacterium prausnitzii* bacteria in all fiber-resistant microbiota, while this microbiota member was not detectable in the identified fiber-sensitive microbiota. Several studies have previously reported a decreased abundance of this bacterium in the colonic mucosa of CD patients with a higher risk of postoperative recurrence, and its anti-inflammatory potential was reported in the chemically-induced colitis model [14, 22]. Moreover, *F. prausnitzii* is considered to be a predominant butyrate producer in the human intestine, together with species belonging to the *Roseburia* genus [23, 24]. Metagenomic analysis confirmed a stark increase in the abundance of *F. prausnitzii*-derived CAzymes (carbohydrate-active enzymes) in fiber-resistant microbiota. These enzymes are key for the fermentation of dietary glycans and the downstream production of short-chain fatty acids (SCFA), thus suggesting that soluble fiber fermentation from *F. prausnitzii* could play a role in mediating the fiber-sensitivity status of a given microbiota. In contrast with this increased abundance of *F. prausnitzii*-derived CAzymes in fiber-resistant microbiota, quantification of these enzymes also revealed increased abundances of CAZymes from other microbiota members, such as *Bacteroides thetaiotaomicron, Bacteroides ovatus, Ruminococcus gnavus* and *Roseburia intestinalis*, in fibre-sensitive microbiota. Hence, this suggest that if microbiota-derived CAZymes arsenal is involved in mediating fibre sensitivity status, it occurs in a species-dependant manner. Moreover, besides dietary glycans fermentation, some of these CAzymes are also involved in mucin-derived glycans degradation [25]. In particular, the literature reports that bacteria such as *A. muciniphila*, *B. thetaiotaomicron* and *B. ovatus* are able to degrade mucin, thus suggesting that they can be involved in the modulation of the intestinal mucosal barrier in a way that could determine microbiota’s degree of sensitivity towards a given soluble fibre.

To conclude, our observations align with previous studies in mice and humans reporting that the addition of fiber to the diet is a “double-edged sword” capable of promoting or demoting gut health in a manner determined not only by the specific fiber being consumed but also based on an individual’s pre-existing microbiota structure and function. Our study not only supports the finding that the extent to which soluble fiber impacts microbiota composition and function is highly donor-dependent, but also suggests that these microbiota-fiber interactions play a cardinal role in modulating intestinal inflammation. In light of the specificity of these microbiota-fiber interactions, further studies appear warranted with the use of a broader selection of soluble fibers in larger cohorts in order to better characterize microbiota sensitivity and the mechanism by which such sensitivity can promote or demote chronic intestinal inflammation. Hence, this study further highlights the importance of personalized fiber-based interventions for patients suffering from IBD. More specifically, our findings suggest the possibility that IBD patients hosting a fiber-resistant microbiota should not refrain from consuming various soluble fibers, while patients with a fiber-sensitive microbiota should carefully consider fiber intake and fiber source as a central actor for disease management. A deeper understanding of the mechanism by which soluble fibers interact with a given intestinal microbiota appears needed to pave the way for the development of personalized fiber- and microbiota-based intervention, both in IBD patients and in healthy individuals.

## Material and methods

### MiniBioReactor Arrays (MBRAs) experiment

#### Fecal sample collection

Fecal samples were provided by six healthy volunteers and collected into sterile containers, sealed, and transferred into an anaerobic chamber within 10 min of defecation. Here, fecal samples were manually homogenized and aliquoted into sterile 50 mL tubes and then stored at − 80 °C until use. The research protocol was approved by the GSU IRB committee under approval number H19174. Individuals donating samples provided informed consent prior to donation.

#### MBRA setup, experimental plan, and timepoints for sample collection

MBRA systems were prepared as previously described [26] and housed in an anaerobic chamber. The system consists of 24 chambers filled with 15 mL of Bioreactor Medium (BRM), as presented in Figure S1A [26]. Chambers were connected to two 24-channel peristaltic pumps with low flow-rate capabilities (205S peristaltic pump with 24-channel drive, Watson-Marlow) and held on a magnetic stand for continual homogenization of the medium. Following autoclaving, MBRA chambers, tubings, and the BRM medium [26] were placed in the anaerobic chambers for at least 72 h. MBRA chambers were subsequently filled with BRM and inoculated with the fecal sample. For the inoculation, fecal samples were resuspended at 10% w/v in anaerobic phosphate-buffered saline (D PBS) (Gibco-Life Technologies) in the anaerobic chamber, vortexed for 5 min, and centrifuged at 800 rpm for 5 min at 20 °C. Supernatants were collected in the anaerobic chamber and filtered through a 100-µm filter in order to remove any particles. The inoculation volume of the fecal slurry was set at 3.8 mL per chamber [26]. After inoculation, fecal microbial communities were allowed to equilibrate for 16 h prior to flow initiation at 1.875 mL/h (8 h retention time). As presented Figure S1B, 0 h timepoint correspond to the inoculation with fecal slurry, and 72 h timepoint correspond to the initiation of fibre treatment (either cellulose, inulin, or psyllium) which lasted until 240 h timepoint. Starting from 0 h, 400 µL of samples were collected at various time points (Figure S1B) and collected samples were stored at − 80 °C until analysis.

### Treatment with fiber: cellulose, inulin, or psyllium

Three independent experiments were performed, with each experiment containing cellulose- (control), inulin-, or psyllium-treated chambers, in triplicates for each of the six donors studies, resulting in a total of nine independent MBRA chambers per donor. The cellulose Solka-Floc® was purchased from Solvaira Specialties. Inulin was purchased from by Sigma Aldrich and psyllium was purchased from J. Rettenmaier & Söhne (https://www.jrs.eu/jrs_en/life-science/food/products/dietary-fibers/). The different fibers were added to the BRM medium prior to autoclaving at a concentration of 0,02%. Bottles with the treatment were connected to the system and used to feed the chamber from 72 to 240 h timepoints. Before the treatment phase (from 0 to 72 h), a regular BRM medium was connected to the system, as presented Figure S1B. Throughout the experiments, BRM-containing bottles were placed on magnetic stands in order to keep constant agitation.

#### Fecal microbiota transplantation to germfree mice, rodent diets used and DSS-induced colitis

Five- or 6-week-old male C57BL/6 germ-free mice (CNRS TAAM UAR44, Orléans, FRANCE) were colonized, upon arrival and under sterile conditions, with human fecal samples from 2 of the donors described above. Briefly, fecal samples from Donors 1 and 2 were diluted in sterile cold PBS at 100 mg/mL, and 200 µL were transferred per mouse per oral gavage. As reported in Figure S5A, two independent Parkbio isolators were used, one per donor, with a total of 3 cages, each containing 5 mice. Mice were acclimatized for 1 week on a purified low-fat diet containing 10% fat, 50 g of cellulose, and 150 g of inulin (Research diet #D190211101, referred to as inulin diet, Table S1). After this acclimatization period aiming to stabilize the donor microbiota within the recipient mice gastrointestinal tract, diets were then pursued on #D190211101 (inulin diet) or switched on either #D13081109 containing 10% fat and 200 g of cellulose (Research Diets, referred as cellulose diet, Table S1) or #D19021103 containing 10% fat, 50 g of cellulose and 150 g of psyllium (Research Diets, referred as psyllium diet, Table S1). After 19 days, colitis was induced in all groups by the addition of DSS in drinking water (2.5% w/v, MP Biomedicals, LCC). Body weights were monitored weekly and fecal samples were collected at various time points throughout the experiment, as presented Figure S6B. After 6 days of DSS exposure, mice were euthanized by cervical dislocation under isoflurane anesthesia. Colon length, colon weight, cecum weight, and spleen weight were measured, and samples were collected for analysis.

#### Fecal flagellin and lipopolysaccharide load quantification

Levels of fecal bioactive flagellin and lipopolysaccharide (LPS) were quantified as previously described [13] using human embryonic kidney (HEK)-Blue-mTLR5 and HEK-Blue-mTLR4 cells, respectively (Invivogen, San Diego, CA, USA) [13]. MBRA samples (whole suspension without centrifugation) were serially diluted and applied to mammalian cells. For fecal samples, fecal material was resuspended in PBS to a final concentration of 100 mg/mL and homogenized for 10 s using a Mini-Beadbeater-24 without the addition of beads to avoid bacteria disruption. Samples were then centrifuged at 8000 × *g* for 2 min and the resulting supernatant was serially diluted and applied to mammalian cells. Purified *E. coli* flagellin and LPS (Sigma-Aldrich) were used for standard curve determination using HEK-Blue-mTLR5 and HEK-Blue-mTLR4 cells, respectively. After overnight incubation at 37 °C, the cell culture supernatant was applied to QUANTI-Blue medium (Invivogen), and the alkaline phosphatase activity was measured at 620 nm after 30 min.

#### Data presentation and statistical analysis

Data are presented as mean ± S.E.M. and significance was determined using one-way group ANOVA with Sidak’s multiple comparisons test (GraphPad Prism software, version 8.0) and differences were noted as significant *p 0.05. As previously reported [9], the following normalizations were applied for select representations of MBRA- and in vivo-based experiments:

- Control group (cellulose-treated) was normalized to 1 for each time point, with inulin- and psyllium-treated groups being expressed as relative values compared to the cellulose-treated group. Through such normalization, the cellulose-treated group is represented as a baseline at a value of 1.

- The last time point before the treatment phase (72 h for the MBRA-based experiment, and D0 for the in vivo-based experiment) was then normalized to 1, for each experimental group, in order to account for pre-treatment variations before fiber manipulation. Through such normalization, all experimental groups start with a value of 1 for the last time point before the treatment phase.

### Supplementary Information

**Additional file 1: Table S1.** Composition of the three purified diets used in this study. Diets used were composed by 10 kcal % of fat and supplemented with 200g per kg of cellulose (cellulose diet), 50g per kg of cellulose +150g per kg of inulin (inulin diet) and 50g per kg on cellulose + 150g per kg of psyllium (psyllium diet). **Table S2.** Composition of the BRM medium used in the in vitro MBRA system. **Figure S1.** Presentation of the MBRA system and schematic outline of the experimental plan used. (A) Overview of the MBRA system installed within an anaerobic chamber and inoculated with human microbiota. (B) Schematic representation of timeline used, samples collected, and analysis performed. **Figure S2.** Efficacy of the MBRA system to reproduce inter-individual variations in microbiota composition. (A) DNA was extracted from MBRA-generated samples collected at the 72h timepoint from chambers inoculated with the 6 human healthy donors used in the study. Microbiota composition was analysed through Illumina-based 16S rRNA gene sequencing. Principal coordinates analysis (PCoA) of the Bray Curtis matrix was computed through the QIIME2 pipeline. Dots are coloured by donor (*N=*9). Significance was determined using non-parametric multivariate analysis of variance (Permanova). (B) Taxonomical composition at the class level of samples collected at the 72h timepoint from the in vitro microbiota MBRA system inoculated with the 6 human healthy donors used in the study, with the 15 most abundant class being represented (*N=*9). (C) Taxonomical composition at the genus level of samples collected at the 72h timepoint from the in vitro microbiota MBRA system inoculated with the 6 human healthy donors used in the study represented (*N=*9). **Figure S3.** Inter-individual variations in fibre-induced metabolomic alterations. The in vitro microbiota MBRA system was inoculated with fecal slurry from 6 healthy donors and stabilized for 72h, at which point fibre treatment was applied using Cellulose, Inulin, or Psyllium. MBRA samples collected during the treatment phase (144h) were used for metabolomic analysis. Principal coordinate analysis of the Bray Curtis distance computed on metabolomic analysis performed on samples collected 72h after the initiation of fibres-treatment are presented. In A, all donors are included, with dots coloured by donor. In B-G, individual donors are represented every 6 donors, with dots coloured by treatment (*N=*3). Significance was determined using non-parametric multivariate analysis of variance (Permanova). **Figure S4.** Inter-individual variations in fibre-induced microbiota metabolomic alterations. The in vitro microbiota MBRA system was inoculated with fecal slurry from 6 healthy donors and stabilized for 72h, at which point fibre treatment was applied using Cellulose, Inulin, or Psyllium. Nineteen metabolites were quantified by HPLC on samples collected 72h after the initiation of fibres-treatment. Data are the means +/- S.E.M, with individual data points being represented (*N=*3). Significance was determined using a one-way ANOVA followed by Tukey’s multiple comparison test and significant differences were recorded as follow: *p<0.05, **p<0.01, ***p<0.001 and ****p<0.0001. **Figure S5.** Inter-individual variations in metagenomic and metatranscriptomic based on the fibre sensitivity status. (A-C) Fecal samples from the donors were used for metagenomic analysis through shotgun sequencing. Obtained quality-filtered reads were grouped *via* MetaPhlAn 2.0 into taxonomical categories and *via* HUMAnN3 into functional categories. (A) Taxonomical features with a statistically significant difference between resistant donors and sensitive donors are presented. (B) CAZymes features with a statistically significant difference between resistant donors and sensitive donors are presented. (C) Relative abundances, for each donor used in this study, of 15 well-known fibre fermenting bacteria. Values are expressed as percentage, and dark blue indicates bacteria that are present in relatively high amount. (D-E) The in vitro microbiota MBRA system was inoculated with fecal slurry from 6 healthy donors and stabilized for 72h, at which point fibre treatment was applied using Cellulose, Inulin, or Psyllium. Total RNAs were extracted from MBRA samples collected during the treatment phase (120h - 144h) and subjected to metatranscriptomic analysis through shotgun sequencing. Obtained quality-filtered reads were grouped *via* HUMAnN3 into functional categories. (C) Principal coordinates analysis (PCoA) of the Bray Curtis distance computed on the generated HUMAnN3 table. All donors are included, and dots are coloured by donor (upper panel) or by treatment (lower panel). (D) HUMAnN3 identified pathway with a statistically significant difference between resistant donors and sensitive donors are presented. **Figure S6.** Schematic representation of the experimental design used for the mice experiement. (A) Upon arrival, germfree C57BL6/J WT mice undergoes fecal microbial transplantation with fecal suspension from donor 1 (*fibres-resistant*) or donor 2 (*fibres-sensitive*) (*N=*15 mice per donor). After one week of microbiota stabilization, mice were subsequently divided into three experimental groups and exposed to either cellulose- (grey), inulin- (purple) or psyllium- (green) supplemented diets for 25 days (*N=*5 mice per experimental group). On day 19 and for 6-days, Dextran Sulphate Sodium was added to the drinking water (2.5% w/v) to induce intestinal inflammation. (B) Schematic representation of timeline used, samples collected, and analysis performed. **Figure S7.** Impact of fibres consumption on intestinal microbiota bacterial load over time. Bacterial DNA was extracted from mice fecal samples and qPCR were performed on 16S rRNA in order to estimate bacterial density. For each donor, bacterial load is expressed as relative value compared to cellulose-treated chambers. Panels A-B represent all the experimental groups in mice transplanted with either donor 1(A) or donor 2 (B) microbiota. Panels C-D represent only inulin-treated (C) or psyllium-treated (D) groups in mice transplanted with either donor. Data presented are the means +/- S.E.M (*N=*5). Significance was determined using 2-way group ANOVA corrected for multiple comparisons with Bonferroni test compared to control group (Cellulose-treated chambers). Significance differences were recorded as follow: **p<0.01 and ****p<0.0001. **Figure S8.** Microbiota composition is differentially impacted by fibre treatment in mice colonized by fibre-resistant and fibre-sensitive donors. Bacterial DNA was extracted from mice fecal samples and subjected to Illumina-based 16S rRNA gene sequencing. (A-D) Beta diversity evolution computed through the QIIME2 pipeline using the Bray Curtis distance matrix. For each donor, evolution of microbiota composition is represented using distances expressed as relative value compared to cellulose-treated mice, defined as 1. Panels A-B represent all the experimental groups in mice transplanted with either donor 1(A) or donor 2 (B) microbiota. Panels C-D represent only inulin-treated (C) or psyllium-treated (D) groups in mice transplanted with either donor. (E-H) Alpha diversity evolution computed through the QIIME2 pipeline using the Evenness index. For each donor, evolution of microbiota composition is represented using distances expressed as relative value compared to cellulose-treated mice, defined as 1. Panels E-F represent all the experimental groups in mice transplanted with either donor 1(E) or donor 2 (F) microbiota. Panels G-H represent only inulin-treated (G) or psyllium-treated (H) groups in mice transplanted with either donor. Data presented are the means +/- S.E.M (*N=*5). Significance was determined using 2-way group ANOVA corrected for multiple comparisons with Bonferroni test compared to control group (Cellulose-treated chambers). Statistical differences were recorded as follow: *p<0.05, **p<0.01, ***p<0.001 and ****p<0.0001. **Figure S9.** Bacterial DNA was extracted from mice fecal samples at day 19 and subjected to Illumina-based 16S rRNA gene sequencing. (A) Taxonomical composition, at the family level, of the fecal microbiota from mice transplanted with donor 1 and treated with either cellulose, inulin or psyllium (*N=*9). (B) Taxonomical composition, at the family level, of the fecal microbiota from mice transplanted with donor 2 and treated with either cellulose, inulin or psyllium (*N=*9). Data are represented as relative abundances (%). The most abundant families are represented, and the terms “others” refers to all families that represented less than 1% of the microbial communities. **Figure S10.** Microbiota pro-inflammatory potential is differentially impacted by fibre treatment in mice colonized by fibre-resistant and fibre-sensitive donors. Microbiota-derived expression of pro-inflammatory molecules lipopolysaccharide (A-D) and flagellin (E-H) were quantified using HEK reporter cells expressing TLR4 or TLR5, respectively. (A-D). Microbiota-derived expression of pro-inflammatory molecules lipopolysaccharide. For each donor, evolution of fecal lipopolysaccharide level is represented as relative value compared to cellulose-treated mice, defined as 1. Panels A-B represent all the experimental groups in mice transplanted with either donor 1(A) or donor 2 (B) microbiota. Panels C-D represent only inulin-treated (C) or psyllium-treated (D) groups in mice transplanted with either donor. (E-H) Microbiota-derived expression of pro-inflammatory molecules flagellin. For each donor, evolution of fecal flagellin level is represented as relative value compared to cellulose-treated mice, defined as 1. Panels E-F represent all the experimental groups in mice transplanted with either donor 1(E) or donor 2 (F) microbiota. Panels G-H represent only inulin-treated (G) or psyllium-treated (H) groups in mice transplanted with either donor. Data presented are the means +/- S.E.M (*N=*5). Significance was determined using 2-way group ANOVA corrected for multiple comparisons with Bonferroni test (# indicates *p<*0.05) compared to control group (Cellulose-treated chambers). Statistical differences were recorded as follow: *p<0.05, **p<0.01, ***p<0.001 and ****p<0.0001. **Figure S11.** Inter-individual variations in fibre-induced body weight modulation. (A-D) Body weight evolution over time expressed as percentage compared to day 0 (start of the fibres treatment), defined as 100%. For each donor, body weight evolution over time is expressed as relative compared to cellulose-treated mice. Panels A-B represent all the experimental groups in mice transplanted with either donor 1(A) or donor 2 (B) microbiota. Panels C-D represent only inulin-treated (C) or psyllium-treated (D) groups in mice transplanted with either donor. Similar representations were used in panels E-H, but only for the DSS-treatment phase. Data presented are the means +/- S.E.M (*N=*5). Significance was determined using 2-way group ANOVA corrected for multiple comparisons with Bonferroni test compared to control group (Cellulose-treated chambers). Statistical differences were recorded as follow: *p<0.05, **p<0.01 and ***p<0.001.

## Data Availability

Unprocessed sequencing data are deposited in the European Nucleotide Archive under accession number PRJEB64164.

## Abbreviations

ASV: Amplicon sequence variant
DSS: Dextran sulfate sodium
FISH: Fluorescent in situ hybridization
FliC: Flagellin
IBD: Inflammatory bowel disease
LPS: Lipopolysaccharide
MBRA: MiniBioReactor Array
PCoA: Principal coordinate analysis
SCFA: Short chain fatty acid
