# Supplementary material for: Individualized microbiotas dictate the impact of dietary fiber on colitis sensitivity
Source: Microbiome. 2024 Jan 5;12:5. doi: 10.1186/s40168-023-01724-6 (PMC10768099; doi:10.1186/s40168-023-01724-6)
Supplement: Supplementary file 2 — Additional file 1: Table S1. Composition of the three purified diets used in this study. Diets used were composed by 10 kcal % of fat and supplemented with 200g per kg of cellulose (cellulose diet), 50g per kg of cellulose +150g per kg of inulin (inulin diet) and 50g per kg on cellulose + 150g per kg of psyllium (psyllium diet). Table S2. Composition of the BRM medium used in the in vitro MBRA system. Figure S1. Presentation of the MBRA system and schematic outline of the experimental plan used. (A) Overview of the MBRA system installed within an anaerobic chamber and inoculated with human microbiota. (B) Schematic representation of timeline used, samples collected, and analysis performed. Figure S2. Efficacy of the MBRA system to reproduce inter-individual variations in microbiota composition. (A) DNA was extracted from MBRA-generated samples collected at the 72h timepoint from chambers inoculated with the 6 human healthy donors used in the study. Microbiota composition was analysed through Illumina-based 16S rRNA gene sequencing. Principal coordinates analysis (PCoA) of the Bray Curtis matrix was computed through the QIIME2 pipeline. Dots are coloured by donor (N=9). Significance was determined using non-parametric multivariate analysis of variance (Permanova). (B) Taxonomical composition at the class level of samples collected at the 72h timepoint from the in vitro microbiota MBRA system inoculated with the 6 human healthy donors used in the study, with the 15 most abundant class being represented (N=9). (C) Taxonomical composition at the genus level of samples collected at the 72h timepoint from the in vitro microbiota MBRA system inoculated with the 6 human healthy donors used in the study represented (N=9). Figure S3. Inter-individual variations in fibre-induced metabolomic alterations. The in vitro microbiota MBRA system was inoculated with fecal slurry from 6 healthy donors and stabilized for 72h, at which point fibre treatment was applied using Cellulose, I [file 40168_2023_1724_MOESM1_ESM.zip › Sup Figures.pdf]

Product #	D13081109		D19021101		D19021103	
	10 kcal% Fat		*10 kcal% Fat*		*10 kcal% Fat*	
	200 g Cellulose		*50 Cell + 150 Inulin*		*50 Cell + 150 Psyllium*	
	gm%	*kcal%*	gm%	*kcal%*	gm%	*kcal%*
Protein	17	*20*	18	*20*	17	*20*
Carbohydrate	59	*70*	70	*70*	58	*70*
Corn Starch	42	*50*	39	*44*	40	*46*
Fat	4	*10*	4	*10*	4	*10*
Total		*100*		*100*		*100*
kcal/gm	3.37		3.53		3.47	
**Ingredient**	**gm**	***kcal***	**gm**	***kcal***	**gm**	***kcal***
Casein	200	*800*	200	*800*	200	*800*
L-Cystine	3	*12*	3	*12*	3	*12*
Corn Starch	506.2	*2025*	450	*1800*	468.7	*1875*
Corn Starch, Hi-Maize 260	0	*0*	0	*0*	0	*0*
Maltodextrin 10	125	*500*	125	*500*	125	*500*
Sucrose	68.8	*275*	68.8	*275*	68.8	*275*
Cellulose, BW200	200	*0*	50	*0*	50	*0*
Inulin, Orafti HP	0	*0*	150	*225*	0	*0*
Pectin, Tic Gums 1400	0	*0*	0	*0*	0	*0*
Psyllium	0	*0*	0	*0*	150	*150*
Lard	20	*180*	20	*180*	20	*180*
Soybean Oil	25	*225*	25	*225*	25	*225*
Mineral Mix S10026	10	*0*	10	*0*	10	*0*
Dicalcium Phosphate	13	*0*	13	*0*	13	*0*
Calcium Carbonate	5.5	*0*	5.5	*0*	5.5	*0*
Potassium Citrate, 1 H2O	16.5	*0*	16.5	*0*	16.5	*0*
Vitamin Mix V10001	10	*40*	10	*40*	10	*40*
Choline Bitartrate	2	*0*	2	*0*	2	*0*
Yellow Dye #5, FD&C	0	*0*	0.05	*0*	0.01	*0*
Red Dye #40, FD&C	0	*0*	0	*0*	0	*0*
Blue Dye #1, FD&C	0.05	*0*	0	*0*	0.04	*0*
**Total**	**1205.05**	***4057***	**1148.85**	***4057***	**1167.55**	***4057***
**gm**						
Total Fiber	200.0		200.0		200.0	
Insoluble Fiber	200.0		50.0		147.5	
Soluble Fiber	0.0		150.0		39.0	
**gm%**						
Total Fiber	16.6		17.4		17.1	
Insoluble Fiber	16.6		4.4		12.6	
Soluble Fiber	0.0		13.1		3.3	

Table S1. Composition of the three purified diets used in this study.

For BRM medium. To 975 mL of distilled water, add:

Quantity	Reagent
1 g	Tryptone
2 g	Proteose peptone
2 g	Yeast extract
100 mg	Arabinogalactan
150 mg	Maltose
150 mg	D-cellobiose
400 mg	Sodium chloride
10 mg	Magnesium sulfate heptahydrate
10 mg	Calcium chloride dihydrate
40 mg	Potassium phosphate dibasic
40 mg	Potassium phosphate monobasic
5 mg	Hemin

For sugar suspension. To 975 mL of distilled water, add:

Quantity	Reagent
25 mL	Distilled water
1 mg	Vitamin K_3
40 mg	D-glucose
200 mg	Inulin
2 g	Sodium bicarbonate

Table S2. Composition of the MBRA medium used for the *in vitro* experiment

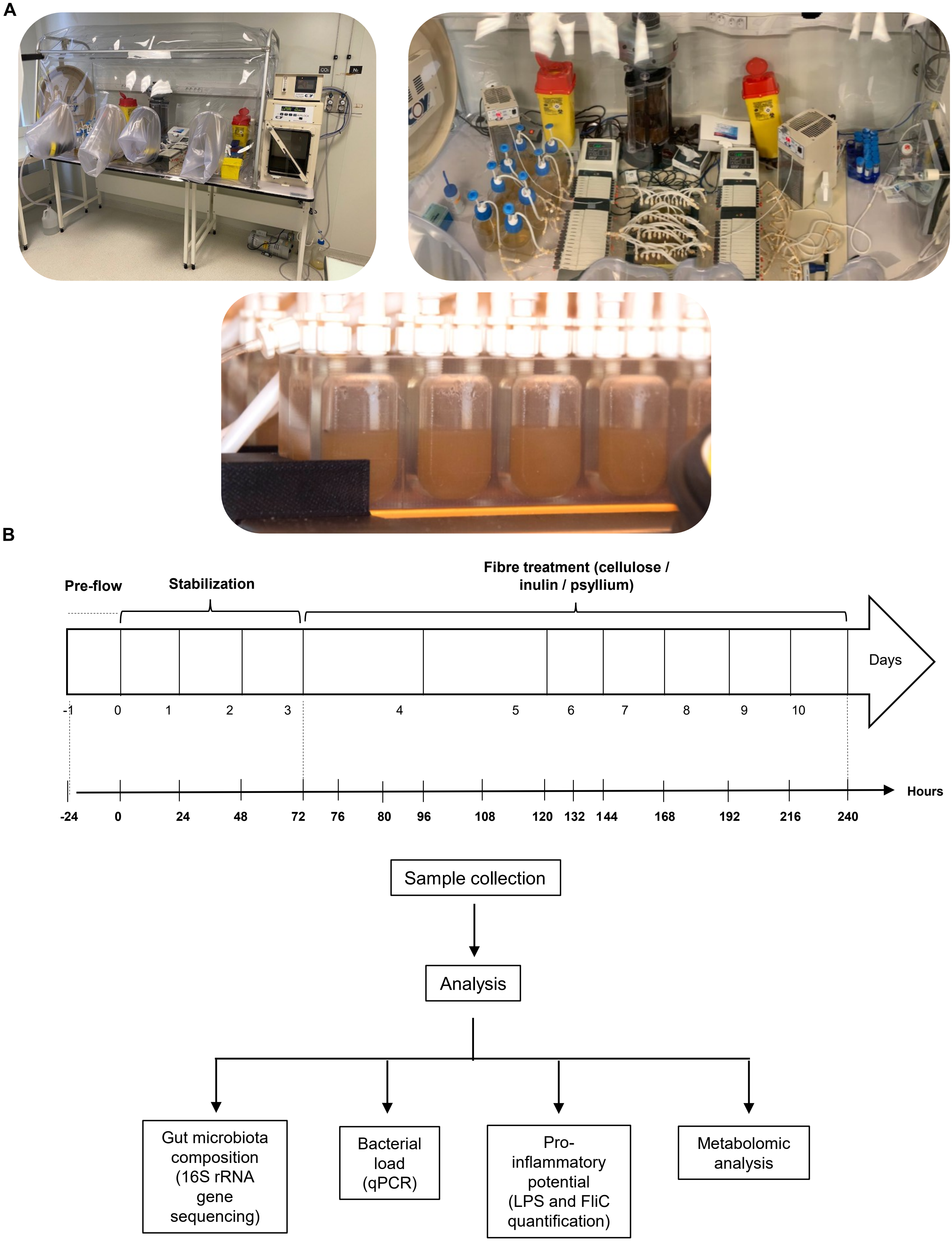

Figure S1. Presentation of the MBRA system and schematic outline of the experimental plan used.

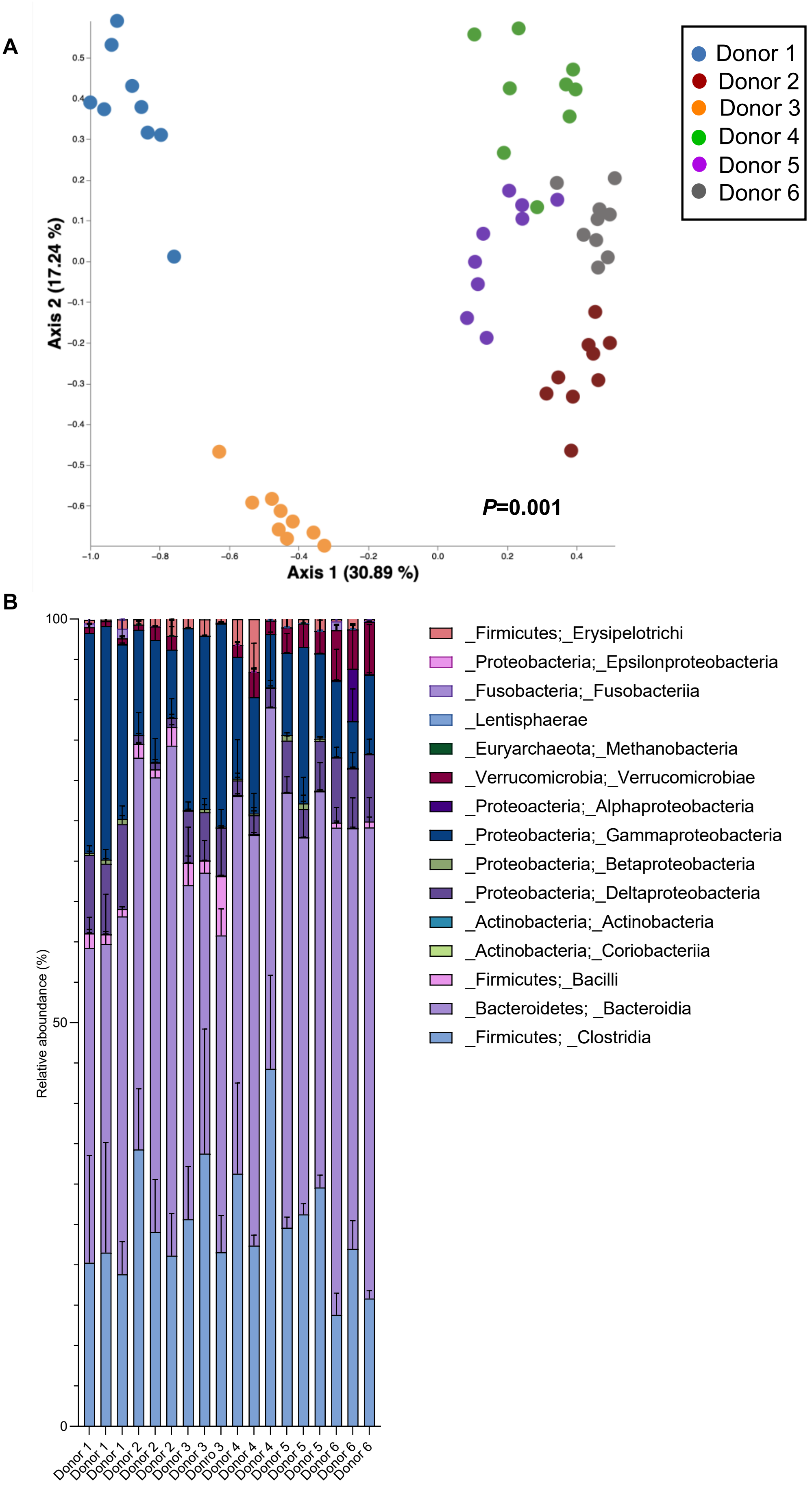

Figure S2. Efficacy of the MBRA system to reproduce inter-individual variations in microbiota composition.

C

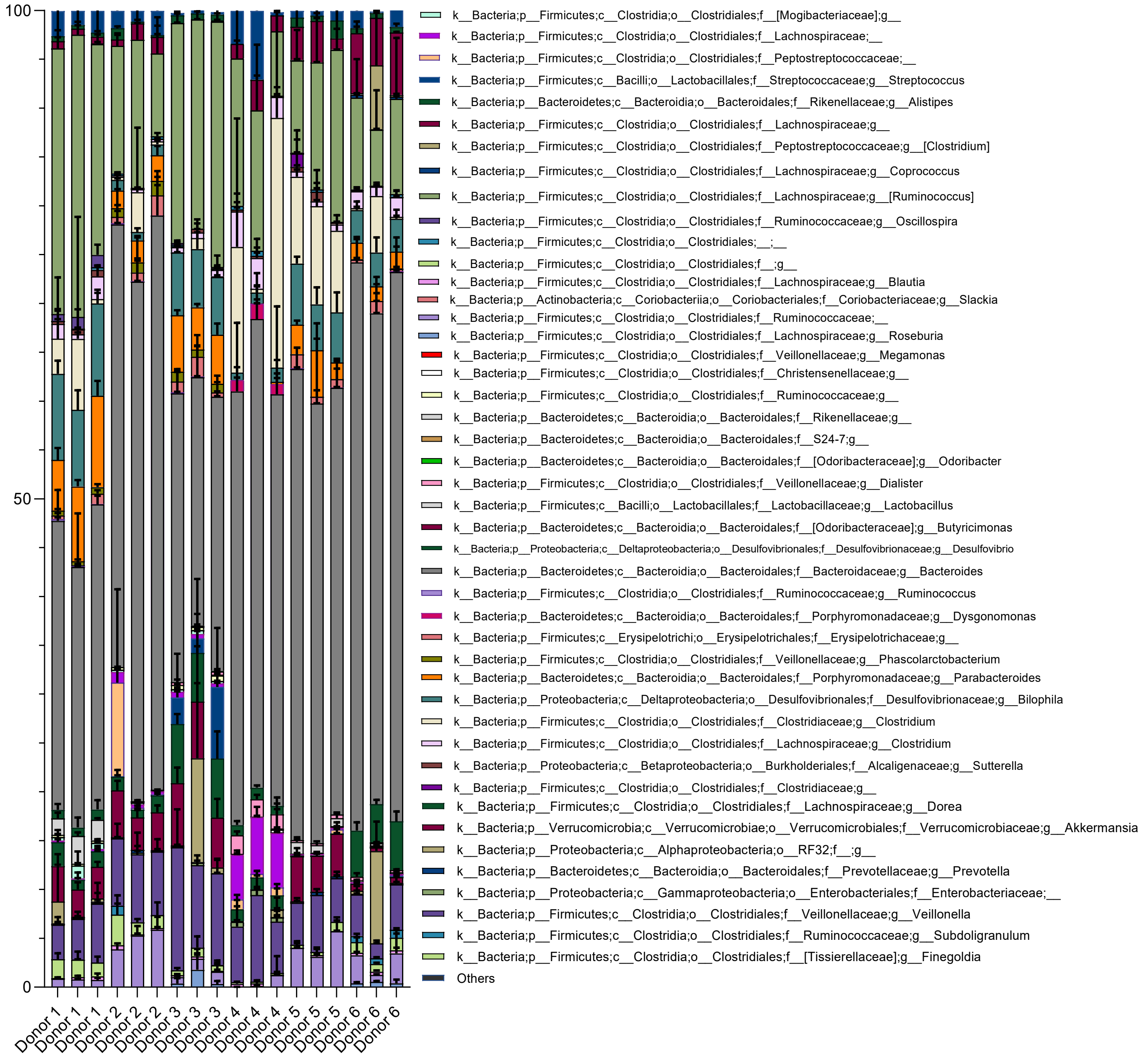

Figure S2. Efficacy of the MBRA system to reproduce inter-individual variations in microbiota composition.

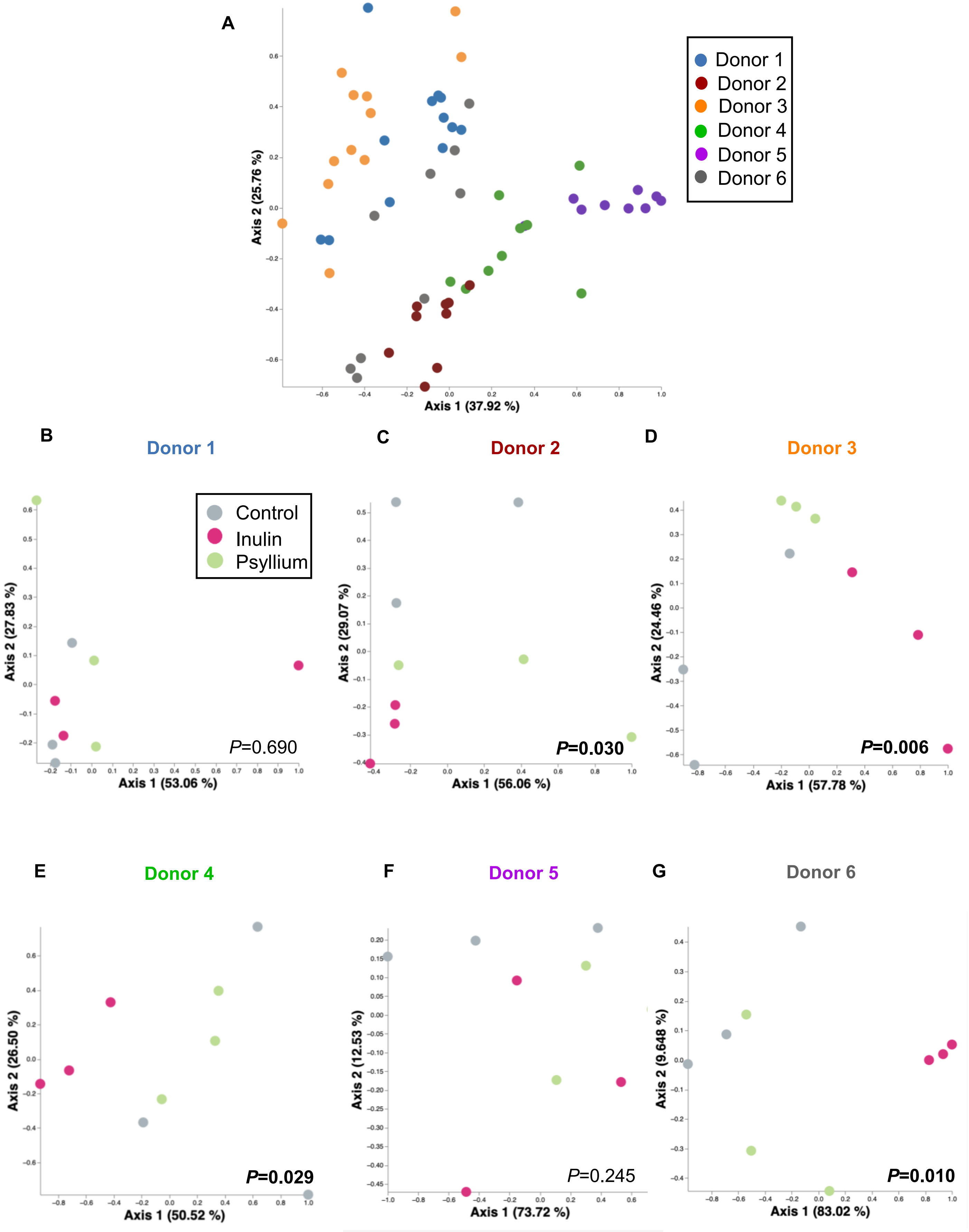

Figure S3. Inter-individual variations in fibres-induced metabolomic alterations.

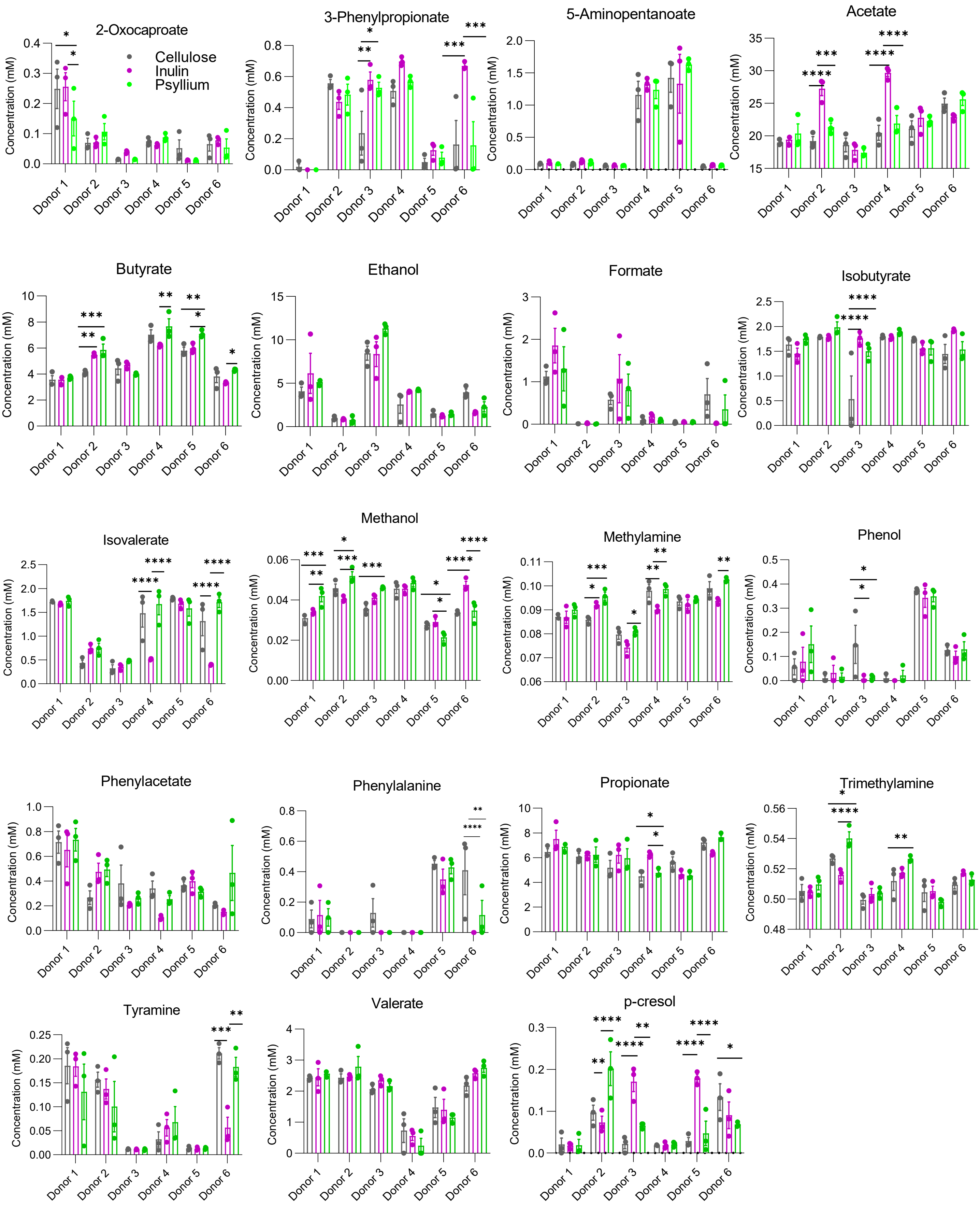

Figure S4. Inter-individual variations in fibres-induced metabolomic alterations.

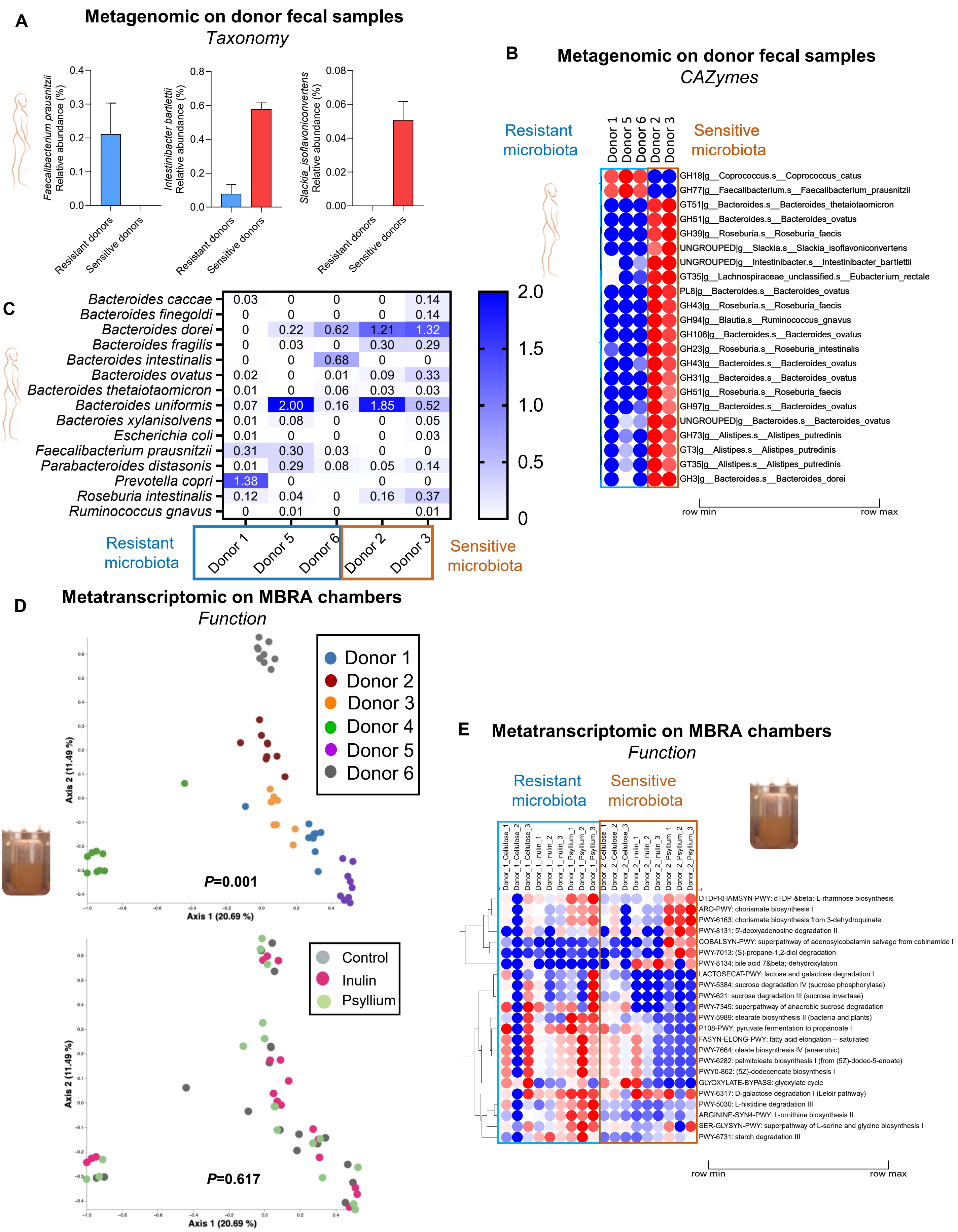

Figure S5. Inter-individual variations in metagenomic and metatranscriptomic based on the fibre sensitivity status.

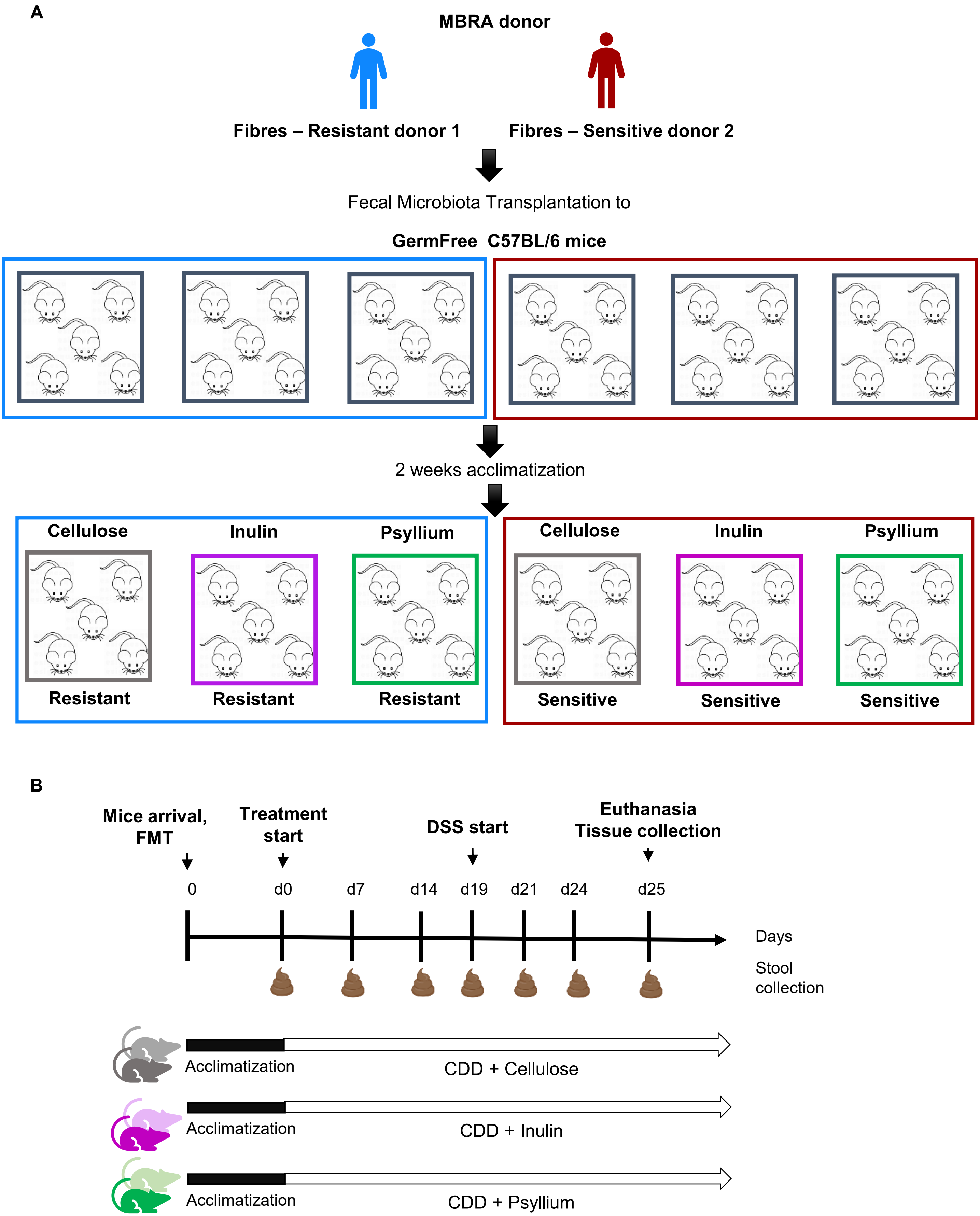

Figure S6. Schematic representation of the experimental design used for the mice experiment.

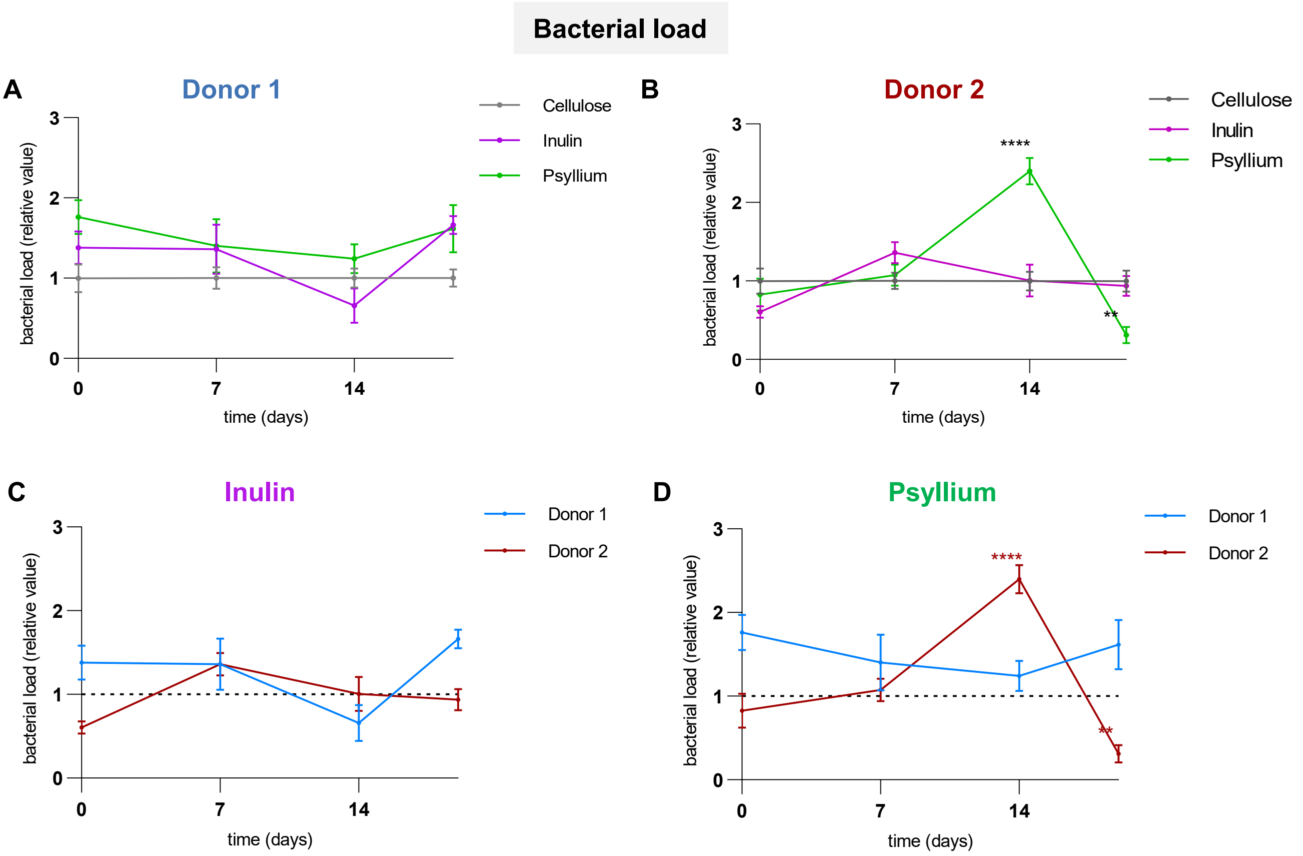

Figure S7. Impact of fibres consumption on intestinal microbiota bacterial load of over time.

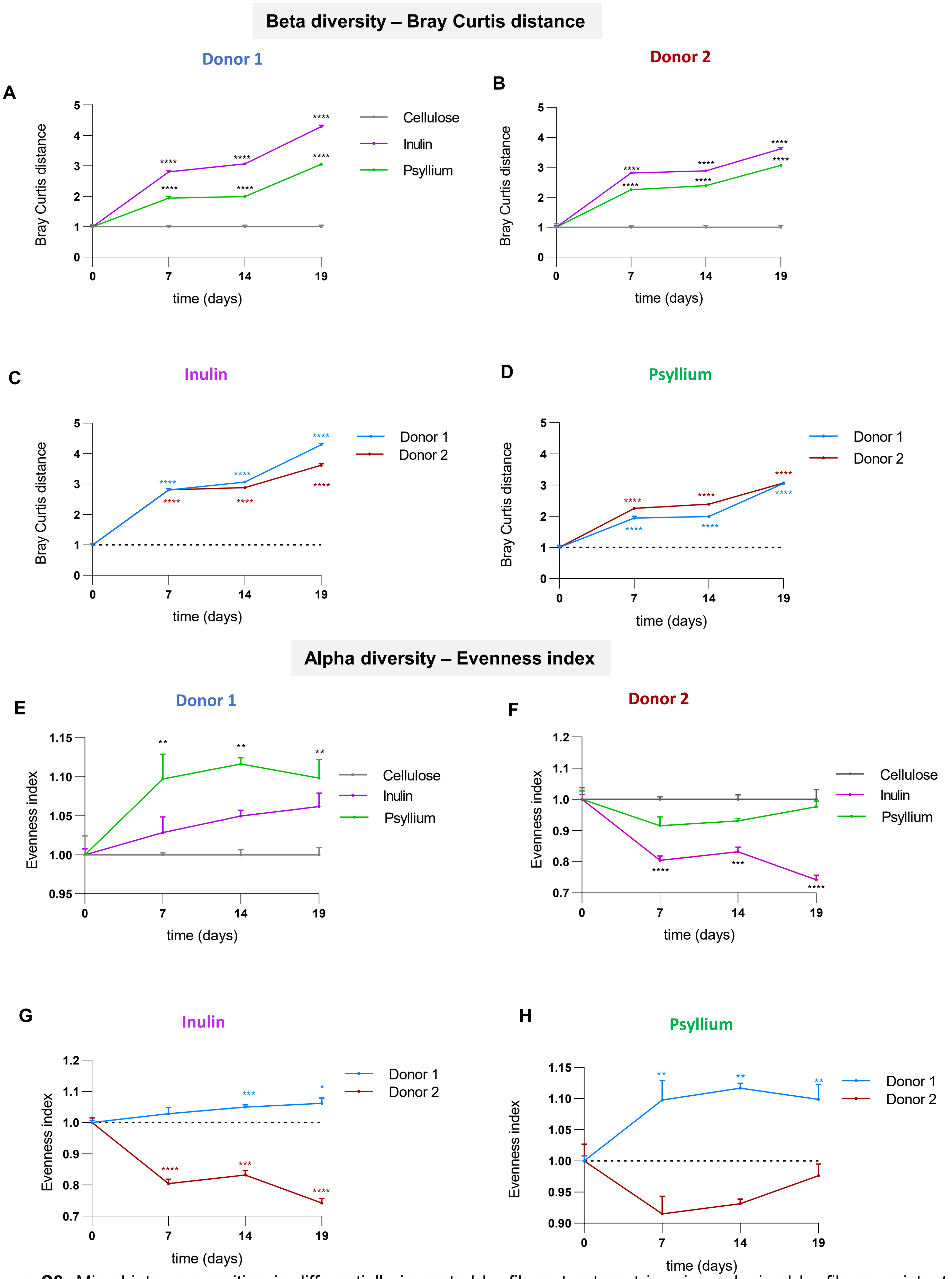

Figure S8. Microbiota composition is differentially impacted by fibres treatment in mice colonized by fibres-resistant and fibres-sensitive donors.

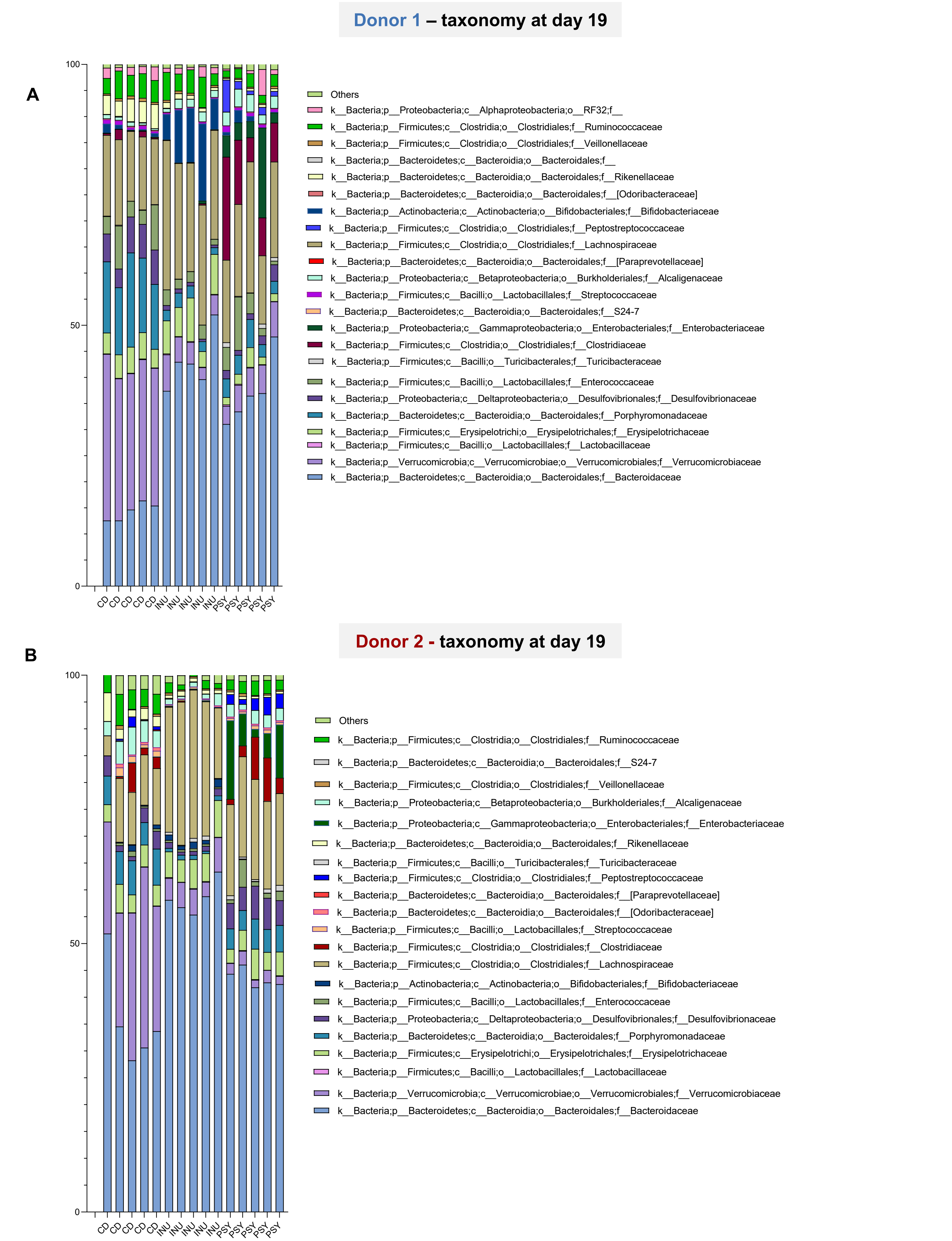

Figure S9. Taxonomic analysis of mice gut microbiota transplanted with Donor 1 and Donor 2 at day 19, prior to DSS exposure

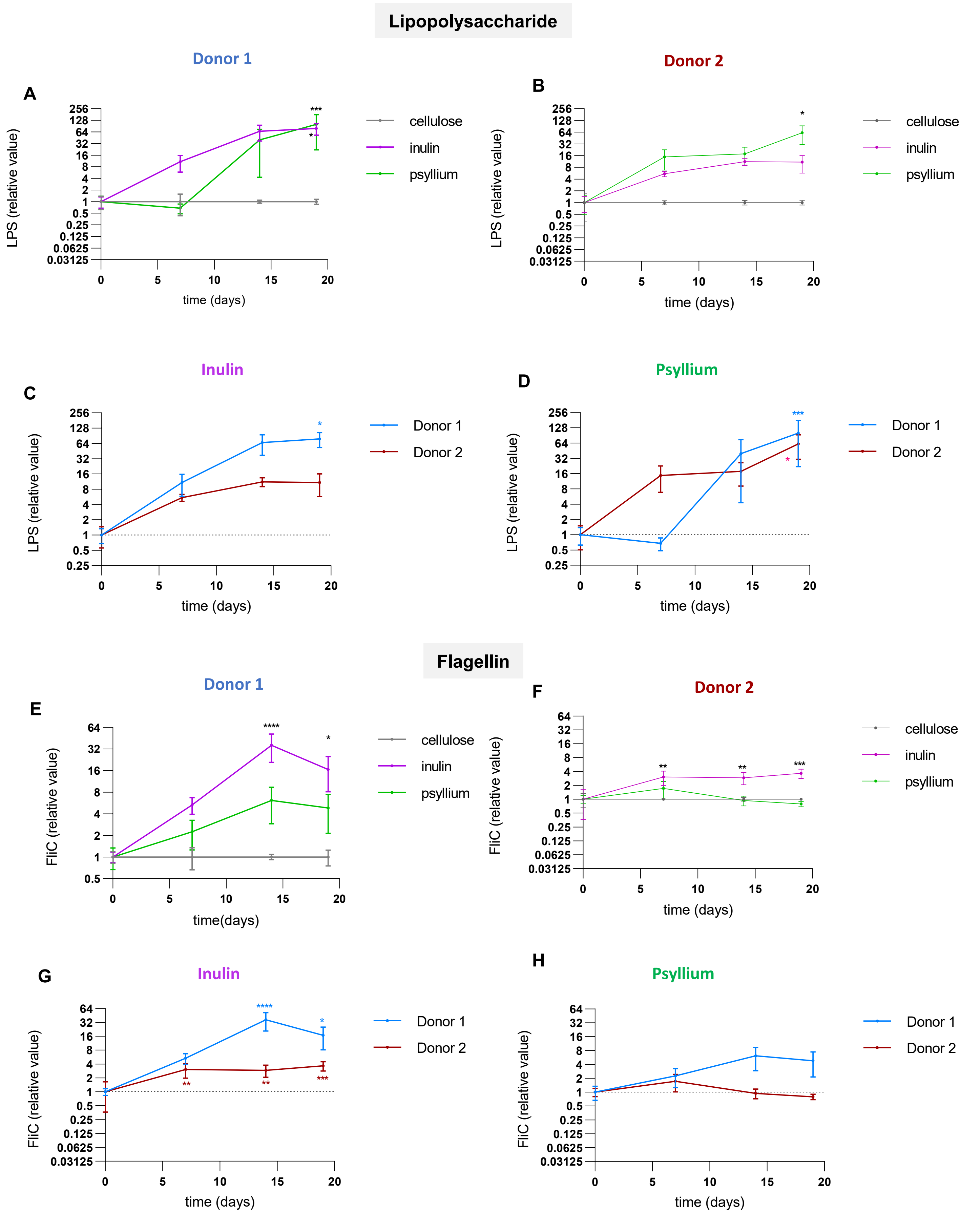

Figure S10. Microbiota pro-inflammatory potential is differentially impacted by fibres treatment in mice colonized by fibres-resistant and fibres-sensitive donors. c

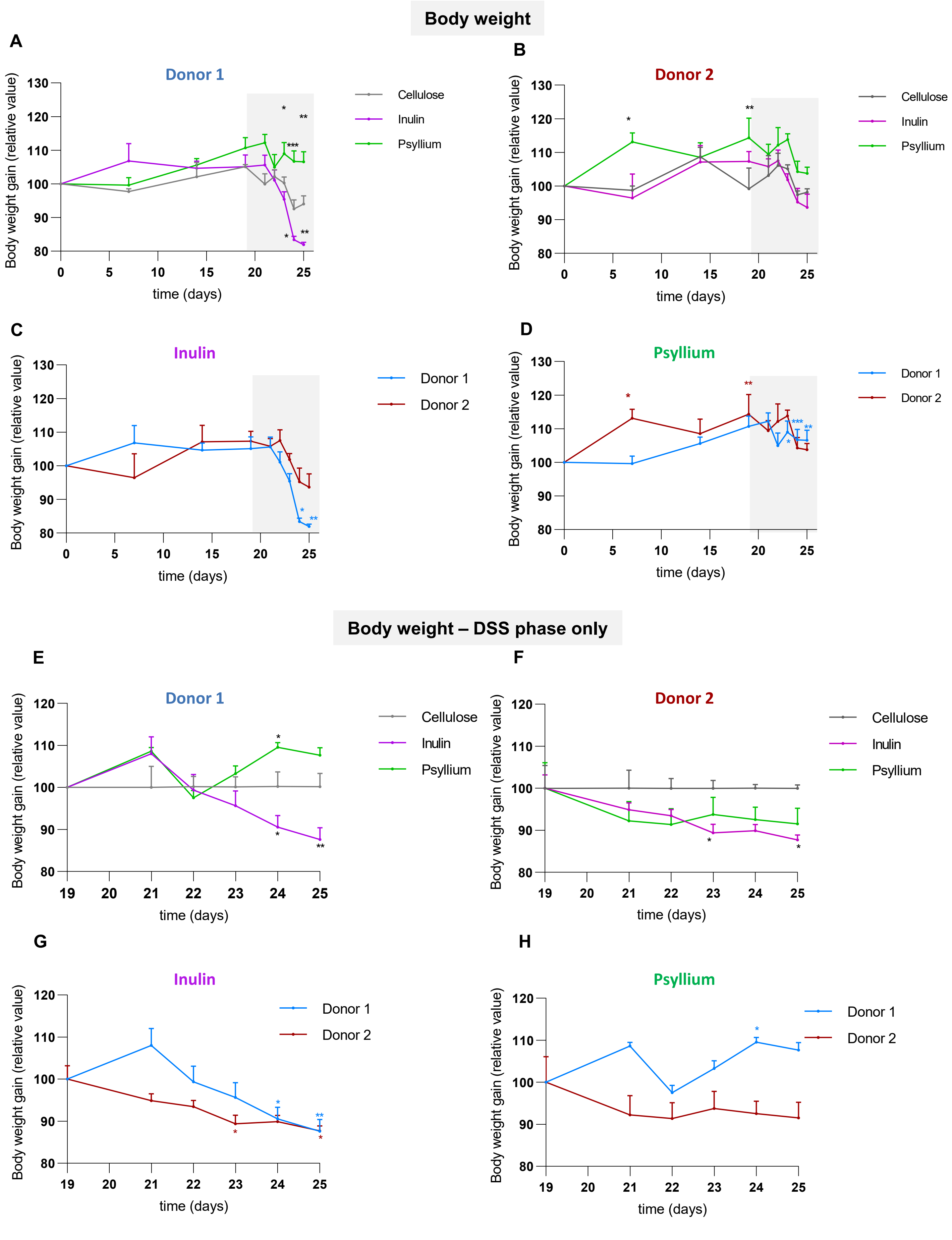

Figure S11. Inter-individual variations in fibres-induced body weight modulation.
