# Supplementary material for: Individualized microbiotas dictate the impact of dietary fiber on colitis sensitivity
Source: Microbiome. 2024 Jan 5;12:5. doi: 10.1186/s40168-023-01724-6 (PMC10768099; doi:10.1186/s40168-023-01724-6)
Supplement: Supplementary file 2 — Additional file 1: Table S1. Composition of the three purified diets used in this study. Diets used were composed by 10 kcal % of fat and supplemented with 200g per kg of cellulose (cellulose diet), 50g per kg of cellulose +150g per kg of inulin (inulin diet) and 50g per kg on cellulose + 150g per kg of psyllium (psyllium diet). Table S2. Composition of the BRM medium used in the in vitro MBRA system. Figure S1. Presentation of the MBRA system and schematic outline of the experimental plan used. (A) Overview of the MBRA system installed within an anaerobic chamber and inoculated with human microbiota. (B) Schematic representation of timeline used, samples collected, and analysis performed. Figure S2. Efficacy of the MBRA system to reproduce inter-individual variations in microbiota composition. (A) DNA was extracted from MBRA-generated samples collected at the 72h timepoint from chambers inoculated with the 6 human healthy donors used in the study. Microbiota composition was analysed through Illumina-based 16S rRNA gene sequencing. Principal coordinates analysis (PCoA) of the Bray Curtis matrix was computed through the QIIME2 pipeline. Dots are coloured by donor (N=9). Significance was determined using non-parametric multivariate analysis of variance (Permanova). (B) Taxonomical composition at the class level of samples collected at the 72h timepoint from the in vitro microbiota MBRA system inoculated with the 6 human healthy donors used in the study, with the 15 most abundant class being represented (N=9). (C) Taxonomical composition at the genus level of samples collected at the 72h timepoint from the in vitro microbiota MBRA system inoculated with the 6 human healthy donors used in the study represented (N=9). Figure S3. Inter-individual variations in fibre-induced metabolomic alterations. The in vitro microbiota MBRA system was inoculated with fecal slurry from 6 healthy donors and stabilized for 72h, at which point fibre treatment was applied using Cellulose, I [file 40168_2023_1724_MOESM1_ESM.zip › Supp text.docx]

**SUPPLEMENTAL MATERIAL AND METHODS**

**Treatment with ceftriaxione antibiotic**

For the study of antibiotic-induced disturbance, similar experimental setup as described above was applied, with a 72h stabilization phase. At this time point and on a daily basis for 5 consecutive days, ceftriaxione was applied as a single injection to reach a final concentration of 100 mg/L.

**Bacterial DNA extraction**

DNA was extracted from frozen MBRA suspension or fecal samples using a QIAmp 96 PowerFecal QIAcube HTkit (Qiagen Laboratories) with mechanical disruption (Qiagen TissueLyser II). Briefly, 650 µL of prewarmed buffer PW1 were added to the samples. Subsequently, samples were thoroughly homogenized using bead-beating with a TissueLyser before centrifuging the plate at 4000rpm for 5 min at 20°C to pellet beads the particles. Four hundred microliters of supernatant were transferred to a new 96-well plate containing 150 µL of buffer C3. After mixing and incubation on ice for 5 min, centrifugation was performed at 4000 rpm for 5 min at 20°C. Three hundred microliters of each supernatant were then transferred to a new 96-well S-block plate and 20 µL of Proteinase K were added and incubated for 10 min at room temperature. The following steps were performed on a QIAcube high-throughput robot as follow: addition of 500 µL of Buffer C4, DNA binding to a QIAmp 96 plate, column wash using 800 µL of AW1, 600 µL of AW2 and 400 µL of ethanol, and elution by adding 100 µL of ATE buffer.

**Bacterial load quantification through 16s rRNA qPCR**

Extracted DNA were diluted 1/10 with sterile DNA-free water and amplified by quantitative PCR using the 16S V4 specific primers 515F 5’-GTGYCAGCMGCCGCGGTAA-3’ and 5’-GGACTACNVGGGTWTCTAAT-3’ on a LightCycler 480 (Roche) using QuantiFAst SYBR® Green Kit (Qiagen). Data were analyzed by the LightCycler® 480 Software, and results were expressed as relative values, per donor, compared to control (cellulose-treated) MBRA chambers.

**Microbiota analysis through 16S rRNA gene sequencing**

16S rRNA gene amplification and sequencing were performed using the Illumina MiSeq technology following the protocol of the Earth Microbiome Project ([www.earthmicrobiome.org/emp-standardprotocols](http://www.earthmicrobiome.org/emp-standardprotocols)) ^1^, with some modifications. Briefly, the 16S rRNA genes, region V4, were PCR amplified from each sample using a composite forward and reverse primer containing a unique 12-base barcode, designed with the Golay error-correcting scheme used to tag PCR products from respective samples ^1^. The forward primer 515F was used 5’-*AATGATACGGCGACCACCGAGATCTACACGCT*XXXXXXXXXXXX**TATGGTAATTGT**GTGYCAGCMGCCGCGGTAA-3’: the italicized sequence is the 5’ Illumina adapter, the 12X sequence is the Golay barcode and the bold sequence is the primer pad, the italicized an bold sequence is the primer linker and the underlined sequence is the conserved bacterial primer 515F. The 806R primer used was 5’-*CAAGCAGAAGACGGCATACGAGAT***AGTCAGCCAGCC**GGACTACNVGGGTWTCTAAT-3’: the italicized sequence is the 3’ reverse complement sequence of Illumina adapter, the bold sequence is the primer pad, the italicized and bold sequence is the primer linker and the underlined sequence is the conserved bacterial primer 806R. PCR reactions consisted of 5PRIME HotMasterMix (Quantabio, Beverly, MA, USA) 0.2 µM of each primer, 10-100 ng template, and reaction conditions were set as follow: 3 min at 95°C, followed by 30 cycles of 45 s at 95°C, 60 s at 50°C and 90 s at 72°C on a Biorad thermocycler. PCR products were then visualized by gel electrophoresis and quantified using Quanti-iT PicoGreen dsDNA assay). A master DNA pool was generated from the purified products in equimolar ratios, and subsequently purified with Ampure magnetic purification beads (Agencourt, Brea, CA, USA). The obtained purified pool was quantified with the Quanti-iT PicoGreen dsDNA assay, followed by sequencing using an Illumina MiSeq sequencer (pair-end reads, 2 x 250 bp) at the GENOM’IC core facility at Cochin Institut, Paris, France. Sequencing depth was 39402 +/- 8141 reads per samples for the MBRA samples and 39533 +/- 10523 reads per samples for the mice samples.

**16S rRNA gene sequence analysis**

QIIME2-version 2022 was used to analyse 16s rRNA sequences^2^. These sequences were demultiplexed and quality filtered using Dada2 method ^3^ with QIIME2 default parameters to detect and correct Illumina amplicon sequence data, generating a table of Qiime 2 artifact. Then, a tree was generated using the align-to-tree-mafft-fasttree command for phylogenetic diversity analysis and we computed alpha and beta diversity analyses using the core-metrics-phylogenetic command. Principal Coordinate Analysis (PCoA) plots were used to assess variations between experimental groups (beta diversity). Alpha diversity was computed with the Evenness index. For the taxonomic analyses, features were assigned to operational taxonomic units (OTUs) with a 99% threshold of pairwise identity to the Greengenes reference database 13_8 ^4^. Unprocessed sequencing data are deposited in the European Nucleotide Archive under accession number PRJEB64164.

**Microbiota analysis through shotgun metagenomic**

Extracted DNAs from the six enrolled donors were mechanically fragmented by sonication using a bath sonicator (Bioruptor® Plus sonication device, Diagenode) 4°C. Library preparation was next performed using the InvitrogenTM ColibriTM PS DNA Library Prep Kit for Illumina® (ThermoFisher) according to manufacturer’s recommendations. A master DNA pool was generated from the purified products in equimolar ratios, and subsequently purified with Ampure magnetic purification beads (Agencourt, Brea, CA, USA). The obtained purified pool was quantified with the Quanti-iT PicoGreen dsDNA assay, followed by sequencing using an Illumina NextSeq500 sequencer (paired-end reads, 2 x 150 bp) at the GENOM’IC core facility at Cochin Institut, Paris, France. Obtained sequences were quality filtered and sequencing adapters were removed from the resulting sequences via cutadapt ^5^. These quality-filtered reads were then grouped *via* MetaPhlAn 2.0 ^6^ into taxonomical categories and *via* HUMAnN3 ^7^ into functional categories.

**Microbiota analysis through shotgun metatranscriptomic**

Total RNAs were extracted from MBRA samples collected during the treatment phase (pool of timepoints 120h and 144h) with the RNeasy PowerMicrobiome Kit (Qiagen), according to the manufacturer’s protocol. RNA concentration and integrity were determined by NanoDropTM Spectrofotometer (Ozyme) and agarose gel electrophoresis, respectively. Total RNAs were then prepared for sequencing using the Illumina® Stranded Total RNA Prep library kit with Ribo-Zero Plus Microbiota rRNA depletion, according to the manufacturer’s protocol. Briefly, rRNAs were depleted using Illumina® Ribo-Zero Plus rRNA Microbiome depletion kit according to the manufacturer’s product for ribodepletion. rRNA-depleted RNAs were then fragmented and converted to cDNA. After end repair and adapters ligation, mRNA libraries were amplified by PCR and validated through BioAnalyser visualization. A master DNA pool was generated from the purified products in equimolar ratios, and subsequently purified with Ampure magnetic purification beads (Agencourt, Brea, CA, USA). The obtained purified pool was quantified with the Quanti-iT PicoGreen dsDNA assay, followed by sequencing using an Illumina NextSeq500 sequencer (single-end reads, 1 x 75 bp) at the GENOM’IC core facility at Cochin Institut, Paris, France. Obtained sequences were quality filtered and sequencing adapters were removed from the resulting sequences via cutadapt ^5^. rRNAs were then removed from the resulting sequences via sortmerna (V2.1) ^8^, and the obtained filtered reads were then grouped *via* HUMAnN3 ^7^ into functional categories.

**Metabolomic analysis**

MBRA samples collected during the treatment phase (144h) were used for metabolomic analysis. Samples preparation for NMR was performed as follow: two hundred microliters of the sample were transferred in a tube where 200 µL of deuterium oxide D_2_O and 60 µL of PBS (Phosphate Buffered Saline) were then added. The samples were vortexed for 10 sec, followed by centrifugation at 17009 g for 10 min at 4°C and subsequently 450 µL of the supernatant were transferred in a 5mm NMR tube for NMR analysis. PCoA plots were used to assess multi-variables variations between experimental groups (beta diversity).

**H&E staining of colonic tissue and histopathologic analysis**

Following euthanasia, 1 cm of the proximal mouse colons were fixed in Carnoy’s fixative solution (60% ethanol, 30% chloroform and 10% glacial acetic acid) and placed tat 4°C until processing. Prior to processing, tissues were washed in methanol 2 x 30 min, ethanol 2 x 15 min, ethanol/xylene (1:1) 15 min and xylene 2 x 15 min, followed by embedding in paraffin. Then, tissues were sectioned at 4-mm thickness and stained with haematoxylin and eosin (H&E) using standard protocols. H&E-stained slides were next scored and each colon was assigned 4 scores based on the degree of epithelial damage and inflammatory infiltrate in the mucosa, submucosa, and muscularis/serosa. The four individual scores per colon were added, resulting in a total scoring range from 0 to 36 per mouse.

**CD68-positive cells staining and quantification**

Staining was performed on the automaton Leica Bond RX. Slides were unmasked at pH6 and incubated 30min with an anti-CD68 antibody (Abcam, EPR23917-164, ab283654) 1:500 diluted and washed. The revelation system (“Bond Polymere Refine” kit, DS9800, Leica) included a secondary antibody HRP conjugated and revealed by DAB. High resolution images were finally acquired using a Lamina Slide Scanner (Perkin Elmer) at the Hist’IM platform (INSERM U1016, Institut Cochin, Paris, France). QuPath (0.3.2 version) was next used to quantify the number of CD68+ intestinal cells per crypt, with 15 crypts over 3 fields being analyzed per mouse.

**REFERENCES**

1. Caporaso, J. G. Ultra-high-throughput microbial community analysis on the Illumina HiSeq and MiSeq platforms. *ISME J.* 4.

2. Bolyen, E. *et al.* Reproducible, interactive, scalable and extensible microbiome data science using QIIME 2. *Nat. Biotechnol.* **37**, 852–857 (2019).

3. Callahan, B. J. *et al.* DADA2: High-resolution sample inference from Illumina amplicon data. *Nat. Methods* **13**, 581–583 (2016).

4. McDonald, D. *et al.* An improved Greengenes taxonomy with explicit ranks for ecological and evolutionary analyses of bacteria and archaea. *ISME J.* **6**, 610–618 (2012).

5. Kechin, A., Boyarskikh, U., Kel, A. & Filipenko, M. cutPrimers: A New Tool for Accurate Cutting of Primers from Reads of Targeted Next Generation Sequencing. *J. Comput. Biol.* **24**, 1138–1143 (2017).

6. Inam, Z. *et al.* Impact of Antibiotics on the Lung Microbiome and Lung Function in Children With Cystic Fibrosis 1 Year After Hospitalization for an Initial Pulmonary Exacerbation. *Open Forum Infect. Dis.* **9**, ofac466 (2022).

7. Beghini, F. *et al.* Integrating taxonomic, functional, and strain-level profiling of diverse microbial communities with bioBakery 3. *eLife* **10**, e65088 (2021).

8. Kopylova, E., Noé, L. & Touzet, H. SortMeRNA: fast and accurate filtering of ribosomal RNAs in metatranscriptomic data. *Bioinformatics* **28**, 3211–3217 (2012).

**SUPPLEMENTAL TABLE LEGEND**

**Table S1. Composition of the three purified diets used in this study.** Diets used were composed by 10 kcal % of fat and supplemented with 200g per kg of cellulose (cellulose diet), 50g per kg of cellulose +150g per kg of inulin (inulin diet) and 50g per kg on cellulose + 150g per kg of psyllium (psyllium diet).

**Table S2.** Composition of the BRM medium used in the *in vitro* MBRA system.

**SUPPLEMENTAL FIGURE LEGENDS**

**Figure S1. Presentation of the MBRA system and schematic outline of the experimental plan used.** (**A**) Overview of the MBRA system installed within an anaerobic chamber and inoculated with human microbiota. (**B**) Schematic representation of timeline used, samples collected, and analysis performed.

**Figure S2. Efficacy of the MBRA system to reproduce inter-individual variations in microbiota composition. (A)** DNA was extracted from MBRA-generated samples collected at the 72h timepoint from chambers inoculated with the 6 human healthy donors used in the study. Microbiota composition was analysed through Illumina-based 16S rRNA gene sequencing. Principal coordinates analysis (PCoA) of the Bray Curtis matrix was computed through the QIIME2 pipeline. Dots are coloured by donor (N=9). Significance was determined using non-parametric multivariate analysis of variance (Permanova). **(B)** Taxonomical composition at the class level of samples collected at the 72h timepoint from the *in vitro* microbiota MBRA system inoculated with the 6 human healthy donors used in the study, with the 15 most abundant class being represented (N=9). (**C**) Taxonomical composition at the genus level of samples collected at the 72h timepoint from the *in vitro* microbiota MBRA system inoculated with the 6 human healthy donors used in the study represented (N=9).

**Figure S3. Inter-individual variations in fibre-induced metabolomic alterations**.

The *in vitro* microbiota MBRA system was inoculated with fecal slurry from 6 healthy donors and stabilized for 72h, at which point fibre treatment was applied using Cellulose, Inulin, or Psyllium. MBRA samples collected during the treatment phase (144h) were used for metabolomic analysis. Principal coordinate analysis of the Bray Curtis distance computed on metabolomic analysis performed on samples collected 72h after the initiation of fibres-treatment are presented. In **A**, all donors are included, with dots coloured by donor. In **B-G**, individual donors are represented every 6 donors, with dots coloured by treatment (N=3). Significance was determined using non-parametric multivariate analysis of variance (Permanova).

**Figure S4. Inter-individual variations in fibre-induced microbiota metabolomic alterations**. The *in vitro* microbiota MBRA system was inoculated with fecal slurry from 6 healthy donors and stabilized for 72h, at which point fibre treatment was applied using Cellulose, Inulin, or Psyllium. Nineteen metabolites were quantified by HPLC on samples collected 72h after the initiation of fibres-treatment. Data are the means +/- S.E.M, with individual data points being represented (N=3). Significance was determined using a one-way ANOVA followed by Tukey’s multiple comparison test and significant differences were recorded as follow: *p<0.05, **p<0.01, ***p<0.001 and ****p<0.0001.

**Figure S5. Inter-individual variations in metagenomic and metatranscriptomic based on the fibre sensitivity status. (A-C)** Fecal samples from the donors were used for metagenomic analysis through shotgun sequencing. Obtained quality-filtered reads were grouped *via* MetaPhlAn 2.0 into taxonomical categories and *via* HUMAnN3 into functional categories. (**A**) Taxonomical features with a statistically significant difference between resistant donors and sensitive donors are presented. (**B**) CAZymes features with a statistically significant difference between resistant donors and sensitive donors are presented. (**C**) Relative abundances, for each donor used in this study, of 15 well-known fibre fermenting bacteria. Values are expressed as percentage, and dark blue indicates bacteria that are present in relatively high amount. (**D-E**) The *in vitro* microbiota MBRA system was inoculated with fecal slurry from 6 healthy donors and stabilized for 72h, at which point fibre treatment was applied using Cellulose, Inulin, or Psyllium. Total RNAs were extracted from MBRA samples collected during the treatment phase (120h - 144h) and subjected to metatranscriptomic analysis through shotgun sequencing. Obtained quality-filtered reads were grouped *via* HUMAnN3 into functional categories. (**C**) Principal coordinates analysis (PCoA) of the Bray Curtis distance computed on the generated HUMAnN3 table. All donors are included, and dots are coloured by donor (upper panel) or by treatment (lower panel). (**D**) HUMAnN3 identified pathway with a statistically significant difference between resistant donors and sensitive donors are presented.

**Figure S6. Schematic representation of the experimental design used for the mice experiement.** (**A**) Upon arrival, germfree C57BL6/J WT mice undergoes fecal microbial transplantation with fecal suspension from donor 1 (*fibres-resistant*) or donor 2 (*fibres-sensitive*) (N=15 mice per donor). After one week of microbiota stabilization, mice were subsequently divided into three experimental groups and exposed to either cellulose- (grey), inulin- (purple) or psyllium- (green) supplemented diets for 25 days (N=5 mice per experimental group). On day 19 and for 6-days, Dextran Sulphate Sodium was added to the drinking water (2.5% w/v) to induce intestinal inflammation. (**B**) Schematic representation of timeline used, samples collected, and analysis performed.

**Figure S7. Impact of fibres consumption on intestinal microbiota bacterial load over time.** Bacterial DNA was extracted from mice fecal samples and qPCR were performed on 16S rRNA in order to estimate bacterial density. For each donor, bacterial load is expressed as relative value compared to cellulose-treated chambers. Panels **A-B** represent all the experimental groups in mice transplanted with either donor 1(**A**) or donor 2 (**B**) microbiota. Panels **C-D** represent only inulin-treated (**C**) or psyllium-treated (**D**) groups in mice transplanted with either donor. Data presented are the means +/- S.E.M (N=5). Significance was determined using 2-way group ANOVA corrected for multiple comparisons with Bonferroni test compared to control group (Cellulose-treated chambers). Significance differences were recorded as follow: **p<0.01 and ****p<0.0001.

**Figure S8. Microbiota composition is differentially impacted by fibre treatment in mice colonized by fibre-resistant and fibre-sensitive donors.** Bacterial DNA was extracted from mice fecal samples and subjected to Illumina-based 16S rRNA gene sequencing. **(A-D)** Beta diversity evolution computed through the QIIME2 pipeline using the Bray Curtis distance matrix. For each donor, evolution of microbiota composition is represented using distances expressed as relative value compared to cellulose-treated mice, defined as 1. Panels **A-B** represent all the experimental groups in mice transplanted with either donor 1(**A**) or donor 2 (**B**) microbiota. Panels **C-D** represent only inulin-treated (**C**) or psyllium-treated (**D**) groups in mice transplanted with either donor. **(E-H)** Alpha diversity evolution computed through the QIIME2 pipeline using the Evenness index. For each donor, evolution of microbiota composition is represented using distances expressed as relative value compared to cellulose-treated mice, defined as 1. Panels **E-F** represent all the experimental groups in mice transplanted with either donor 1(**E**) or donor 2 (**F**) microbiota. Panels **G-H** represent only inulin-treated (**G**) or psyllium-treated (**H**) groups in mice transplanted with either donor. Data presented are the means +/- S.E.M (N=5). Significance was determined using 2-way group ANOVA corrected for multiple comparisons with Bonferroni test compared to control group (Cellulose-treated chambers). Statistical differences were recorded as follow: *p<0.05, **p<0.01, ***p<0.001 and ****p<0.0001.

**Figure S9.** Bacterial DNA was extracted from mice fecal samples at day 19 and subjected to Illumina-based 16S rRNA gene sequencing. (**A**) Taxonomical composition, at the family level, of the fecal microbiota from mice transplanted with donor 1 and treated with either cellulose, inulin or psyllium (N=9). (**B**) Taxonomical composition, at the family level, of the fecal microbiota from mice transplanted with donor 2 and treated with either cellulose, inulin or psyllium (N=9). Data are represented as relative abundances (%). The most abundant families are represented, and the terms “others” refers to all families that represented less than 1% of the microbial communities.

**Figure S10. Microbiota pro-inflammatory potential is differentially impacted by fibre treatment in mice colonized by fibre-resistant and fibre-sensitive donors.** Microbiota-derived expression of pro-inflammatory molecules lipopolysaccharide (**A-D**) and flagellin (**E-H**) were quantified using HEK reporter cells expressing TLR4 or TLR5, respectively. **(A-D)**. Microbiota-derived expression of pro-inflammatory molecules lipopolysaccharide. For each donor, evolution of fecal lipopolysaccharide level is represented as relative value compared to cellulose-treated mice, defined as 1. Panels **A-B** represent all the experimental groups in mice transplanted with either donor 1(**A**) or donor 2 (**B**) microbiota. Panels **C-D** represent only inulin-treated (**C**) or psyllium-treated (**D**) groups in mice transplanted with either donor. **(E-H)** Microbiota-derived expression of pro-inflammatory molecules flagellin. For each donor, evolution of fecal flagellin level is represented as relative value compared to cellulose-treated mice, defined as 1. Panels **E-F** represent all the experimental groups in mice transplanted with either donor 1(**E**) or donor 2 (**F**) microbiota. Panels **G-H** represent only inulin-treated (**G**) or psyllium-treated (**H**) groups in mice transplanted with either donor. Data presented are the means +/- S.E.M (N=5). Significance was determined using 2-way group ANOVA corrected for multiple comparisons with Bonferroni test (# indicates p<0.05) compared to control group (Cellulose-treated chambers). Statistical differences were recorded as follow: *p<0.05, **p<0.01, ***p<0.001 and ****p<0.0001.

**Figure S11. Inter-individual variations in fibre-induced body weight modulation.** (**A-D**) Body weight evolution over time expressed as percentage compared to day 0 (start of the fibres treatment), defined as 100%. For each donor, body weight evolution over time is expressed as relative compared to cellulose-treated mice. Panels **A-B** represent all the experimental groups in mice transplanted with either donor 1(**A**) or donor 2 (**B**) microbiota. Panels **C-D** represent only inulin-treated (**C**) or psyllium-treated (**D**) groups in mice transplanted with either donor. Similar representations were used in panels **E-H**, but only for the DSS-treatment phase. Data presented are the means +/- S.E.M (N=5). Significance was determined using 2-way group ANOVA corrected for multiple comparisons with Bonferroni test compared to control group (Cellulose-treated chambers). Statistical differences were recorded as follow: *p<0.05, **p<0.01 and ***p<0.001.
